# Genome-wide gene expression analysis suggests an important regulatory role of lncRNAs in primary Sjögren’s syndrome

**DOI:** 10.3389/fimmu.2026.1751195

**Published:** 2026-04-15

**Authors:** Zhongshan Li, Ping Wang, Jiazheng Wang, Hui Cheng, Yue Chen, Lihe Zhang, Xiaofang Zhu, Wenyu Chen, Jingwei Hong, Yujuan Wang, Jinyu Wu

**Affiliations:** 1 Institute of Genomic Medicine, Wenzhou Medical University, Wenzhou, China; 2 Department of Rheumatology and Immunology, Changzheng Hospital, Naval Medical University, Shanghai, China; 3 Department of Rheumatology, The Second Affiliated Hospital of Wenzhou Medical University, Wenzhou, China; 4 Department of Rheumatology, The First Affiliated Hospital of Wenzhou Medical University, Wenzhou, China; 5 Department of Geriatrics, Lishui People’s Hospital, Lishui, China

**Keywords:** ceRNA network, lncRNA, minor salivary gland, primary Sjögren’s syndrome, total transcriptome sequencing

## Abstract

**Introduction:**

Primary Sjögren’s syndrome (pSS) is a common autoimmune disease, with the minor salivary gland (MSG) being the main affected tissue; however, its pathogenesis remains unclear. Although changes in long noncoding RNA (lncRNA) expression have been reported in pSS, their biological functions are uncertain, despite the regulatory roles suggested. Therefore, it would be meaningful to systematically investigate the gene expression of lncRNAs in pSS for their regulatory roles and possible contribution to the disease development.

**Methods:**

Deep stranded total transcriptome sequencing was performed on MSG samples from 92 patients with pSS and 34 non-Sjögren’s syndrome (non-SS) controls. Differentially expressed genes between pSS and non-SS were identified, and a genome-wide competing endogenous RNA (ceRNA) network was constructed based on shared miRNA binding sites and gene–expression correlations. The regulatory roles of lncRNAs in dysregulated pathways in pSS were assessed by examining expression changes of interacting lncRNAs and coding genes. *In vitro* overexpression experiments in a salivary gland cell line were performed to evaluate the regulatory roles of three selected lncRNAs as ceRNAs.

**Results:**

Genome-wide coding and noncoding gene expression correlation analysis suggests the regulatory function of lncRNAs in pSS. LncRNA could regulate coding gene expression via ceRNA mechanisms. The genome-wide ceRNA network comprising 3,035 lncRNAs and 10,838 coding genes was constructed. Eight lncRNAs were predicted to play essential roles on coding gene expression changes in pSS. The regulatory effect for three of the eight key lncRNAs—BISPR, LINC00926, and HCP5—were validated by *in vitro* experiment and their expression was correlated with clinical features of pSS.

**Discussion:**

Systematic analysis of coding and lncRNA expression in MSG samples suggests a genome-wide regulatory role for lncRNAs in pSS. We constructed, for the first time, a genome-wide ceRNA network. This ceRNA network can be used to infer lncRNA functions based on their interacting coding genes. The gene regulatory roles of three lncRNAs were validated. Our study suggests that lncRNAs contribute significantly to coding gene expression changes in pSS via ceRNA mechanisms, and the identified regulatory lncRNA candidates could be useful for diagnosis, sub-classification, and treatment.

## Introduction

Primary Sjögren’s syndrome (pSS) is one common type of autoimmune disease in which the minor salivary glands (MSGs) are the main affected tissues, typically showing lymphocytic infiltration (1–4). The etiology of pSS is poorly understood, and environmental, genetic, or epigenetic factors could contribute to its onset and progression (4, 5). Genome-wide association studies (GWAS) of pSS have identified many associated genetic loci (6–8), most of which lie in intergenic regions, which indicates that the disease could be caused by gene expression changes. Gene expression in pSS has been analyzed in many studies using microarray or RNA sequencing (RNA-seq), and genes in innate or adaptive immune processes, such as interferon (IFN) cytokine pathways, were frequently found to be dysregulated (8–10). In addition, gene expression profiles have been used for pSS diagnosis (11), prognosis (12), and subtyping (9, 10). However, the mechanisms underlying coding gene expression changes in pSS remain unclear, which is essential for understanding the disease biogenesis.

It is of note that most studies of pSS mainly focus on coding genes, and noncoding RNA genes were less studied because of lack of function characterizations (13, 14). Accumulating evidence suggests that long noncoding RNAs (lncRNAs) play significant roles on the pathogenesis of autoimmune diseases including rheumatoid arthritis (RA), systemic lupus erythematosus (SLE), and systemic sclerosis (SSc) (8, 15–17). In pSS, studies of gene expression in blood (18–24) and MSG (25, 26) have found a lot of disease-associated lncRNAs, such as BISPR, NEAT1, TMEVPG1, and PVT1. It is of note that some of the lncRNAs could regulate pathways related to pSS biogenesis, for example, PVT1 on CD4^+^ T-cell activation (22); LINC01871 on IFNγ stimulation and T-cell activation (19); and NEAT1 on TNF-α and MAPK pathways (24). However, the functions of most lncRNAs are unclear, despite the general understanding that lncRNAs often act as regulators by binding DNA, RNA, or proteins (14, 27). For example, studies in cancer have shown that lncRNAs could bind miRNAs as “competing endogenous RNA” (ceRNA) to regulate coding gene expression (28–31). In fact, lncRNAs functioning as ceRNAs have also been described in many autoimmune diseases (32–36). In pSS, to our knowledge, analysis of lncRNAs as ceRNAs was performed in only one study by Chen (20), wherein total transcriptome sequencing of peripheral blood mononuclear cells (PBMCs) in 45 patients with pSS and healthy subjects was analyzed to identify the ceRNA network for four selected lncRNAs. Therefore, it is essential to evaluate the regulatory functions of lncRNAs in pSS from the perspective of ceRNA mechanisms.

Actual ceRNA identification requires experimental evidence such as crosslinking immunoprecipitation (HITS-CLIP) (37, 38), which is time-consuming and labor-intensive. Given the competitive binding of miRNAs to lncRNAs and mRNAs at shared recognition sites, it is reasonable and efficient to predict ceRNA networks from gene expression correlations, as carried out in many studies (34, 39–45). For example, in Tay’s study of the PTEN gene, expression correlation was found with its predicted ceRNAs, and the regulation effects were validated in the cell line using siRNA-mediated knockdown experiment (45); Luo et al. predicted the ceRNA pair NLRP3–lncRNA4344 from expression correlations and shared miRNA binding and validated the regulatory relationship experimentally (46). In Zhang’s work, the expression data of 12 cancers from the TCGA database were obtained to calculate lncRNA–mRNA expression correlations for ceRNA filtering (39). In Xu’ s work, based on shared miRNA binding site prediction, positive expression correlation was used to filter ceRNA interactions in 20 cancers (43). In our work, we aim to construct a whole genome ceRNA network based on expression correlation between all possible lncRNA–mRNA pairs with shared miRNA binding in MSG of pSS. The deep total transcriptome RNA sequencing was applied to MSG samples from 92 patients with pSS and 34 non-Sjögren’s syndrome (non-SS) subjects to measure expression for both coding and noncoding genes genome-widely. To our knowledge, this is the largest study of gene expression using total transcriptome sequencing in the mainly affected tissue of pSS. We have found evidence for the significant regulatory role of lncRNAs on coding gene expression and pSS-related pathway activities. Our work deepens our understanding of pSS biogenesis from the perspective of lncRNA regulation, and these findings could promote the disease diagnosis, prognosis, and treatment.

## Materials and methods

### Patient cohorts

A total of 126 subjects presenting with dry mouth symptoms but without any known autoimmune disease diagnoses were recruited from the Wenzhou Medical University, Wenzhou, China (**Table 1**). All subjects were of Han Chinese ethnicity and aged between 20 and 60 years, with the majority being female. Clinical features relevant to Sjögren’s syndrome were collected for each subject, including assessments for anti-SSA, anti-SSB antibodies, gland atrophy, and lymphocytic infiltration. Among the 126 subjects, 92 were diagnosed with pSS according to the 2016 ACR-EULAR classification criteria, and the remaining 34 subjects who did not meet the diagnostic criteria were classified as non-SS (controls). A summary of the participants’ information is presented in **Table 1**. The study was approved by the Medical Ethical Committee of Wenzhou Medical University and written informed consent was obtained from all participants.

**Table 1:** Clinical characteristics for study cohorts.

Characteristic	pSS(92)	non-SS(34)	Test p value	Significance
Gender	87/92(95%)	28/34(82%)	0.07>	No
Dry mouth	45/90(50%)	14/33(42%)	0.54	No
Dry eye	30/91(31%)	11/33(33%)	1	No
anti-SSA	63/91(69%)	4/33(12%)	1.01*10-8	Yes
anti-SSB	30/90(33%)	3/34(9%)	0.006	Yes
anti-ANA	50/90(56%)	19/33(58%)	1	No
anti-Ro	56/89(63%)	4/33(12%)	4.13*10-7	Yes
Glander wither	78/86(91%)	6/24(18%)	1.34*10-14	Yes
Lymphacyte infiltration	45/86(52%)	1/34(3%)	6.6*10-8	Yes
Age	49.72	52.94	0.23	No
IgG	18.67	12.88	3.42*10-6	Yes

Data are presented as n/N (%) for categorical variables: n represents the number of subjects with the characteristic present and N represents the total number of subjects with available data for that feature. Continuous variables (Age and IgG) are presented as mean values.

The p-values were calculated using Fisher’s exact test for categorical variables and the Student’s t-test for continuous variables.

Significance of statistical test was defined as Yes if p value <= 0.05 and No if >0.05.

Abbr: pSS, primary Sjögren’s syndrome; non-SS, non-Sjögren’s syndrome controls; anti-SSA, anti-Sjögren’s-syndrome-related antigen A autoantibodies; anti-SSB, anti-Sjögren’s-syndrome-related antigen B autoantibodies; anti-ANA, anti-nuclear antibodies; anti-Ro, anti-Ro autoantibodies; IgG, immunoglobulin G.

### Sample collection, cDNA library construction, and sequencing

The MSG samples were obtained from the inner surface of the lower lip. The dissected samples were paraffin-embedded, sectioned, and stained with hematoxylin and eosin by experienced pathologists. Biopsy samples were snap-frozen and stored in liquid nitrogen until RNA extraction. Total RNA was extracted using the RNeasy Mini Kit according to the manufacturer’s instructions (Qiagen). The RNA Integrity Number (RIN) of the samples was assessed using an Agilent Bioanalyzer 2100 (Agilent Technologies, USA) and a NanoDrop ND-2000 spectrophotometer to evaluate RNA integrity. Only samples meeting quality thresholds (RIN ≥ 7.0 and 28S/18S ratio ≥ 0.7) were included for transcriptome sequencing. A sequencing library was constructed using the TrueSeq RNA Sample Preparation Kit (Illumina). The concentration of the libraries was measured using a Qubit^®^ 2.0 Fluorometer, and RNA fragment size was assessed on an Agilent 4200. High-throughput sequencing for cDNA was performed on an Illumina MiSeq platform.

### RNA-seq data processing and differentially expressed gene detection

Stranded total transcriptome sequencing was performed on the 126 samples and processed through quality control, trimming, alignment, and assembly. Gene expression levels in TPM and exonic read counts for both known and novel assembled genes were estimated using RNA-SeQC (v2.4.2). Log2-transformed and quantile-normalized TPM values were utilized to represent gene expression level during analysis. The CIBERSORTx (https://cibersortx.stanford.edu/, absolute mode) tool was used to estimate immune cells’ percent for each sample using the LM22 signature matrix. The gene expression was adjusted for total immune cell percent predicted using the R/Bioconductor package limma. Differentially expressed genes (DEGs) between patients with pSS and non-SS subjects were identified using the DESeq2 software (v1.44), with sequencing batch and immune cell percent as covariates.

### Gene expression correlation calculation

Pairwise expression (adjusted TPM) correlations between coding genes and lncRNA genes were calculated across all salivary gland samples. LncRNA–coding gene pairs with Pearson correlation coefficients greater than 0.7 or less than −0.7, and Bonferroni-corrected *p*-values less than 0.05, were considered to be significantly correlated. For comparison, gene expression (TPM) correlations were also calculated for 161 healthy individuals downloaded from the GTEx (v8) database.

### Genome-wide ceRNA network construction

The ceRNA interactions between coding genes and lncRNA genes were inferred based on shared miRNA binding sites and significant positive expression correlations. A total of 668 miRNA with expression levels greater than 1 TPM in the MSG were downloaded from the miTED database and used for ceRNA interaction analysis here. The miRNA–lncRNA binding data were obtained from the ENCORI and NPInter databases. Experimental data for miRNA–mRNA (coding genes) binding were obtained from ENCORI, miRTarBase, and TarBase V8. This yielded a total of 2,208,961 interactions between 2,842 miRNAs and 17,925 coding genes. Additionally, predicted miRNA-coding gene binding was acquired from TargetScan V8 and miRDB. As a result, we retained 429,771 high-confidence miRNA–mRNA interactions (supported by both experimental and predicted evidence), involving 2,489 miRNAs and 14,702 coding genes. LncRNA–mRNA pairs that shared miRNA bindings for the expressed miRNAs and showed significant positive expression correlation (Pearson *r* > 0.5, Bonferroni-corrected *p* < 0.05) were selected as putative ceRNA interactions (see **Supplementary Methods** for further details).

### Pathway activity estimates

Gene set variation analysis (GSVA) was employed to estimate pathway activity. Briefly, normalized gene expression levels were used as input for GSVA in R to produce gene set-level scores that reflect pathway activity. Gene sets for 1,692 REACTOME pathways were downloaded from the MSigDB database (https://www.gsea-msigdb.org/gsea/msigdb). The Student’s *t*-test was utilized to compare gene set scores between pSS and non-SS samples, and the thresholds of false discovery rate (FDR) < 0.05 and absolute fold change (FC) ≥ 2 were used to call differentially expressed pathways.

### A253 cell culture and lentiviral plasmids infection

The A253 cell line was obtained from the American Type Culture Collection (ATCC), stored, and processed according to the manufacturer’s instructions. Briefly, cells were stored at temperatures below −130 °C, preferably in liquid nitrogen vapor. The cell lines were authenticated through STR profiling and confirmed to be mycoplasma negative. All cells were cultured in DMEM (Gibco) supplemented with 10% FBS at 37 °C in a 5% CO_2_ atmosphere.

Lentiviral plasmids expressing the human lncRNAs BISPR, LINC00926, and HCP5 were purchased from Genechem (Shanghai, China), and the RNA interference target sequences were obtained from the GENCODE database. The recombinant lentiviral vector was constructed by cloning the overexpression sequences into the pGCSIL-green fluorescent protein lentivirus vector using AgeI/EcoRI restriction sites.

### Transcriptome sequencing of A253 cell line

The lncRNAs were overexpressed (OE) via lentiviral infection in A253 to produce three biological replicates for each, and three empty-vector infection replicates were produced as negative control (NC). Standard transcriptome sequencing (RNA-seq) was performed on each replicate, and the reads were processed to obtain gene-level expression (see Materials and Methods). The three lncRNAs were successfully transfected into the A253 cell lines with >90% efficiency. DEGs between three OE replicates and three NC replicates were identified using DEseq2 software (adjusted *p*-value < 0.05 and absolute fold change >2) for each lncRNA separately.

To validate the regulatory effects of the three lncRNAs as ceRNA, expression changes between OE and NC cell lines for predicted interacting genes (on ceRNA network constructed) for the lncRNAs were compared with other genes. To validate regulatory effects of lncRNAs on gene expression changes in pSS, DEGs detected in pSS vs. non-SS samples were compared with DEGs detected in lncRNA OE vs. NC cell lines.

Please see **Supplementary Methods** for further details about data processing and analysis.

## Results

### Study cohorts

A cohort of 126 subjects presenting with sicca symptoms and without any known autoimmune diseases were enrolled in this study and all were of Han Chinese ethnicity. Comprehensive clinical characteristics related to pSS were collected for all participants. Of all subjects, 92 were diagnosed as pSS according to the 2016 ACR-EULAR classification criteria (47), and the remaining 34 were used as control (non-SS). The MSG tissue samples were collected for each subject. See **Table 1** and Methods for details.

### Genome-wide transcript detection in minor salivary glands

**Figure 1:**
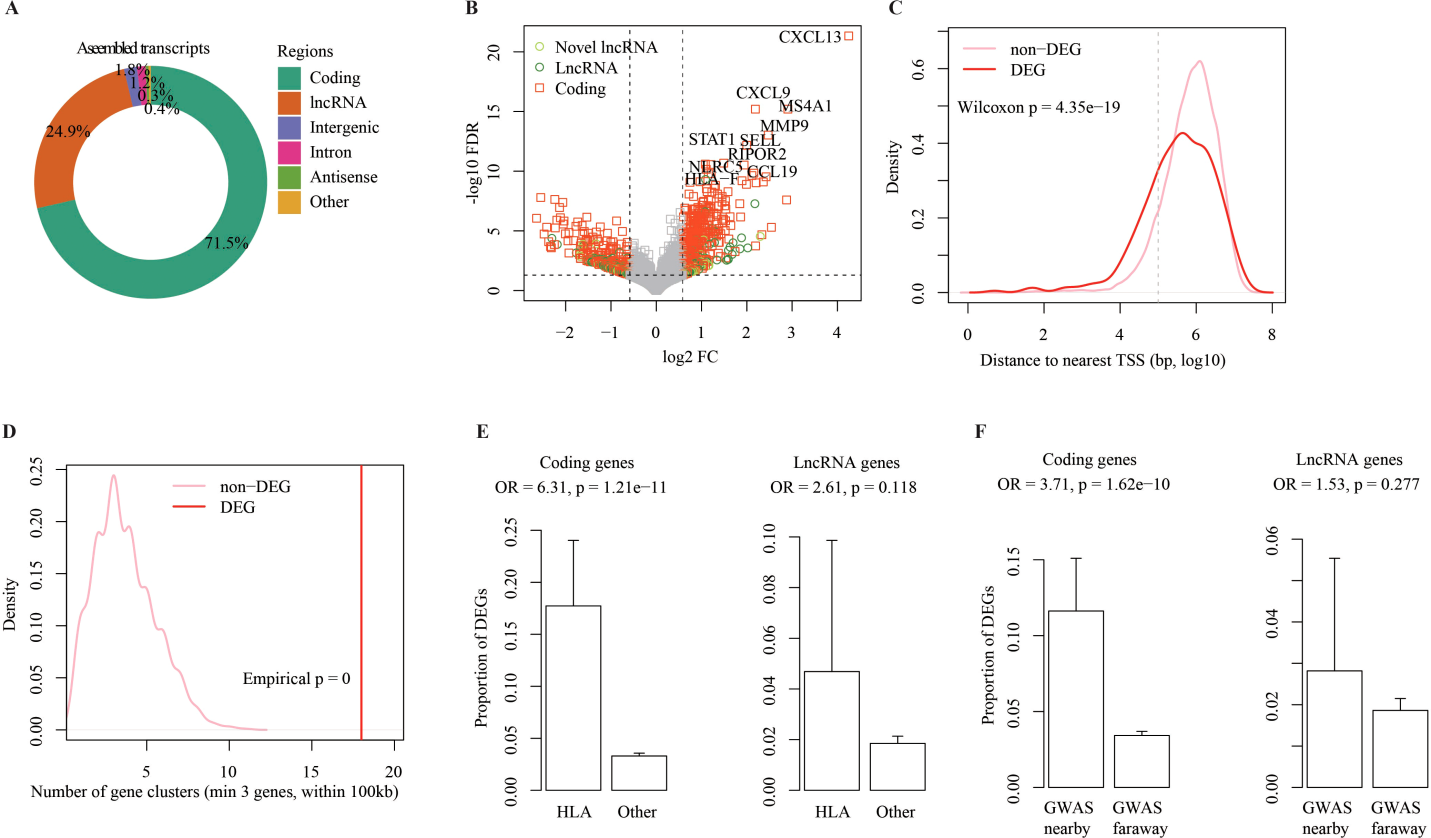
Genome-wide identification of differentially expressed genes in pSS vs non-SS. **(A)** Assembled transcripts in different genome regions. Different genome regions based on the Genecode annotations (v46 GRCH38) were labeled using different colors. Other represents transcripts from rRNA, snoRNA, or tRNAs. **(B)** Volcano plot to show differentially expressed (DE) genes detected in pSS vs. non-SS. Log-transformed fold change (FC) and adjusted *p*-values (FDR) are shown on the x-axis and y-axis. Dashed lines label FC of 1.5 (vertical) and FDR of 0.05 (horizontal). Different colors/shapes are for different biotypes and gray is for non-significant differentially expressed genes. **(C)** Distribution of distances between nearby genes for pSS DEGs and non-DEGs separately. The distances were log 10 transformed, and the dashed line represents 100 kbp distance. The brick red line is for pSS DEGs and the pink line is for non-DEGs. **(D)** Distribution of gene cluster counts detected among pSS DEGs and non-DEGs. The vertical brick red line labels count of gene clusters detected among 790 pSS DEGs. The pink density line represents distribution of gene cluster counts detected among equal-sized non-DEGs by 1,000 times random sub-sampling of 27,295 non-DEGs. Empirical *p*-value for observed DEG clusters was labeled. **(E)** Proportion of pSS DEGs detected in HLA and other genome regions. Left for coding genes and right for lncRNA genes. Bar height indicates the proportion of DEGs among all expressed genes in the region and error bar for 95% confidence interval is shown. Enrichment odds ratio and one-sided Fisher’s exact test *p*-value are labeled on top. HLA region: chr6:28,510,120-33,480,577 on hg38 based on NCBI definitions. **(F)** Proportion of pSS DEGs detected in pSS association genome loci. Left for coding genes and right for lncRNA genes. Bar height for proportion of DEGs nearby pSS association locus (≤100 kbp) or far away (>100 kbp) with error bar for 95% confidence interval. Enrichment odds ratio and one-sided Fisher’s exact test *p*-value are labeled on top.

Deep stranded total transcriptome sequencing was performed on the 126 MSG samples, generating approximately 40–70 million 101-bp paired-end reads each (**Supplementary Table 1A**). *De novo* transcript assembly of sequencing reads identified 233,631 unique long transcripts (≥2 exons and ≥200 bp long) across the 126 samples. The majority of assembled transcripts originated from annotated coding regions (166,941, 71.5%, Genecode, v109) and lncRNA (58,222, 24.9%), with few (7,672, 3.2%) from intron, antisense, or intergenic regions (**Figure 1A**; **Supplementary Table 1B**). Ultimately, a total of 17,369 known coding genes, 10,267 known lncRNA genes, and 2,485 novel lncRNA genes were obtained. Transcripts for three novel lncRNAs were validated using RT-PCR and Sanger sequencing in three independent MSG samples (**Supplementary Figure 1**, see **Supplementary Methods**).

### Differentially expressed genes detected in pSS and non-SS

Analysis of gene expression level across samples has revealed that coding genes generally exhibited much higher expression than lncRNA genes, but lncRNA expression was more variable (**Supplementary Figure 2A**). Principal component analysis (PCA) has revealed separation between pSS and non-SS samples (**Supplementary Figure 2B**). Genome-wide identification of DEGs between pSS and non-SS samples has found 790 DEGs (**Figure 1B**, absolute fold change ≥ 1.5 and adjusted *p* < 0.05; **Supplementary Table 2** and Methods), which include 578 known coding genes (3.4%), 158 known lncRNA genes (1.9%), and 54 novel lncRNA genes (2.29%) (**Supplementary Figure 2C**).

To verify our findings, 4,035 coding and 60 lncRNA genes with expression significantly changed in pSS compared with control (fold change ≥2 and adjusted *p* < 0.05) were collected from publications (**Supplementary Table 1C**). As a result, among 578 pSS DE coding genes detected here, 375 (64%) were reported by previous studies. As for 158 DEGs of lncRNA genes, 9 (5.6%) were reported previously. Both overlaps are statistically significant (**Supplementary Figure 2D**). Gene Ontology (GO) term enrichment analysis for the DE coding genes revealed that upregulated genes in pSS were primarily related to immune system activations (**Supplementary Figure 2E**), whereas downregulated genes were mainly involved in epidermal functions (**Supplementary Figure 2F**).

The pSS DEGs were found to be non-uniformly distributed along the genome from global view (**Supplementary Figures 3A,B**). The distances between nearby pSS DEGs on genome were significantly smaller than between non-DEGs (**Figure 1C**). Eighteen clusters (containing 73 genes) were found among the known DEGs, but only two to four clusters were expected among equal-sized non-DEGs (**Figure 1D**, **Supplementary Figure 3C**). The Gene Set Enrichment Analysis (GSEA) identified seven chromosome cytogenetic bands with significantly larger expression changes in pSS vs. non-SS (**Supplementary Figure 3D**). Interestingly, the pSS-associated region HLA (48, 49) was found to show significant enrichment of pSS DEGs (**Figure 1E**, see Methods). Further analysis has found that pSS DEGs is significantly enriched among the 156 associated genes from pSS GWAS studies (**Supplementary Table 3**; **Supplementary Figure 3E**) and in nearby genomic regions (**Figure 1F**).

### Gene expression correlation analysis suggests regulatory effects of lncRNAs

**Figure 2:**
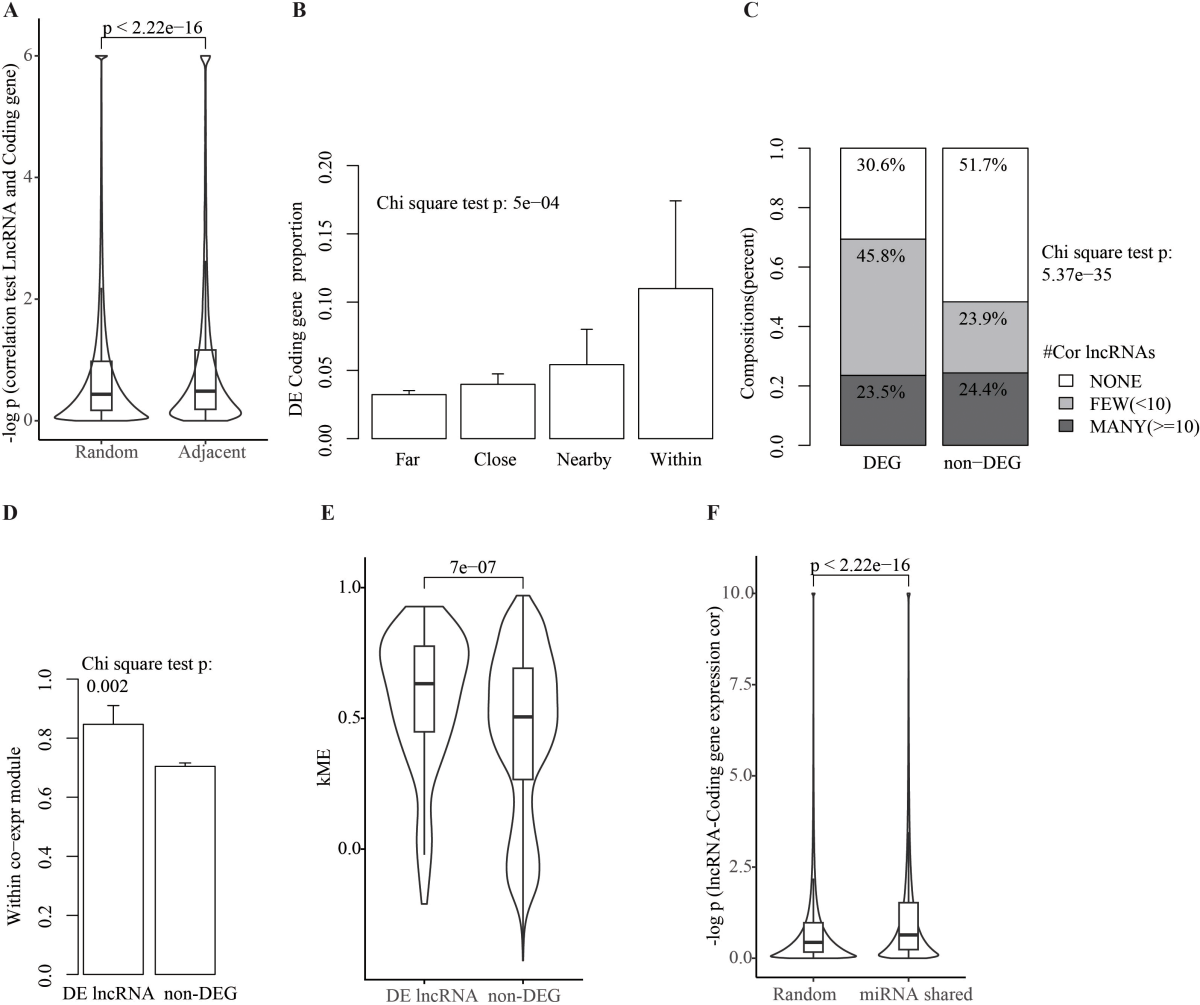
Coding and lncRNA gene expression correlation and expression changes in pSS vs. non-SS. **(A)** Higher expression correlation between adjacent lncRNA and coding genes pairs than random gene pairs. The *y*-axis is for −log10-transformed *p*-value from gene expression correlation test (Pearson). Wilcoxon rank test was used for comparison of expression correlation test *p*-values between adjacent (overlapping or TSS distances <100 kbp) lncRNA–coding gene pairs and random gene pairs, with *p*-value labeled on top. **(B)** Distribution of pSS DE coding genes with varying distances to pSS DE lncRNAs. Coding genes expressed in pSS were split into four groups based on distance (TSS) to nearest pSS DE lncRNA: within (gene body overlap, 91 genes), nearby (<100 kbp, 295 genes), close (100–500 kbp, 2,536 genes), and far (>500 kbp, 13,970). Bar height represents pSS DE gene proportion among all coding genes within each bin, with error bar for 95% confidence interval labeled. The chi-square test was used to test the gene enrichment pattern, and *p*-values are shown. **(C)** More correlated lncRNAs for pSS DE coding genes. Coding genes are divided into three groups based on the number of correlated lncRNA genes: 0 (none), 1–10 (few), and >10 (many). Proportion of genes with different numbers of correlated lncRNA for DE and non-DE coding genes are shown as stacked bar plot. Chi-square test *p*-values are shown. **(D)** Proportion of lncRNAs within gene co-expression modules. The bar’s height represents the proportion of lncRNAs that were included in any co-expression modules among all expressed lncRNAs, and was shown for DE lncRNAs and non-DE lncRNAs separately. The error bar for 95% confidence interval was shown. The proportion differences between DE lncRNAs and non-DE lncRNAs were tested using the chi-square test and *p*-value shown. The 58 co-expression modules were detected among all expressed genes across MSG samples using the WGCNA software (see Methods for details). **(E)** Comparison of module membership for pSS DE and non-DE lncRNAs. The module membership for each gene within gene co-expression module was measured as kME (WGCNA), with higher kME as hub. The two-sided Wilcoxon rank test was used for differences test of kME between the two groups, with *p*-value labeled on top. **(F)** Comparison of expression correlation between lncRNA and coding genes with shared miRNA binding sites or not. The *y*-axis is for log10-transformed *p*-value from test of expression correlation (Pearson). The Wilcoxon rank test was used for comparison of expression correlation *p*-values between the two groups, and the resultant *p*-value was labeled on top.

Clustering of DEGs shown above indicates that gene expression in pSS could be regulated by local genetic elements on chromosome. It was found that expression correlations between adjacent lncRNA and coding genes (overlapping or TSS distances <100 kbp) were significantly higher than between random gene pairs (**Figure 2A**). In addition, the proportion of pSS DE coding genes is much higher among genes within or nearby pSS DE lncRNA genes than those far away (**Figure 2B**). These results propose possible regulation of lncRNA on nearby genes in pSS, and it is essential to investigate lncRNA regulation on a genome-wide scale.

To systematically estimate lncRNA regulatory effects on mRNA, the co-expression between all paired lncRNA and coding genes was calculated. Considering that immune infiltration is common in MSG of pSS samples and could affect gene expression measure for bulked RNA-seq as used here, the immune cell proportion was estimated using CIBERSORTx (https://cibersortx.stanford.edu/) and corrected before expression correlation calculation (see Materials and Methods). As a result, a total of 681,694 significant correlations (absolute Pearson correlation coefficients of ≥0.7 and adjusted *p*-value < 0.05) were detected between the 8,510 coding genes and 3,427 lncRNA genes. Notably, most lncRNA and coding genes are positively rather than negatively correlated (57.6%, binomial test *p*-value < 2.2e−16), which is consistent with the ceRNA interaction mechanism.

To investigate the relationship between gene expression correlation and expressing changes in pSS vs. non-SS, it was found that among pSS DE coding genes, 69.4% genes with ≥1 correlated lncRNAs was found, which is significantly higher than 48.3% among non-DE coding genes (chi-square test *p*: 5.37e−35, **Figure 2C**). Notably, when lncRNA–coding gene expression correlations were calculated using normal MSG samples from the Gtex project, more correlated lncRNAs for the pSS DEGs than non-DEGs were observed similarly (**Supplementary Figure 3F**). In another way to investigate the regulatory role of lncRNA, the gene co-expression network in pSS was constructed using the R package WGCNA (50 co-expression modules comprising 22,112 genes, **Supplementary Figure 3G**) to estimate lncRNA’s regulatory role using network connectivity. Firstly, it was found that significantly more pSS DE lncRNAs were assigned to any of the co-expression modules than non-DE lncRNAs (83% vs. 64%, **Figure 2D**). Then, for lncRNA within co-expression modules, the connectivity (kME by WGCNA) for DE lncRNA genes is significantly higher than for non-DE lncRNAs (**Figure 2E**), which means pSS DE lncRNA’s tendency to become hub genes or key regulators. Overall, the above analysis suggests that coding gene expression changes in pSS could be regulated by lncRNAs.

### Genome-wide lncRNA–mRNA ceRNA network prediction

**Figure 3:**
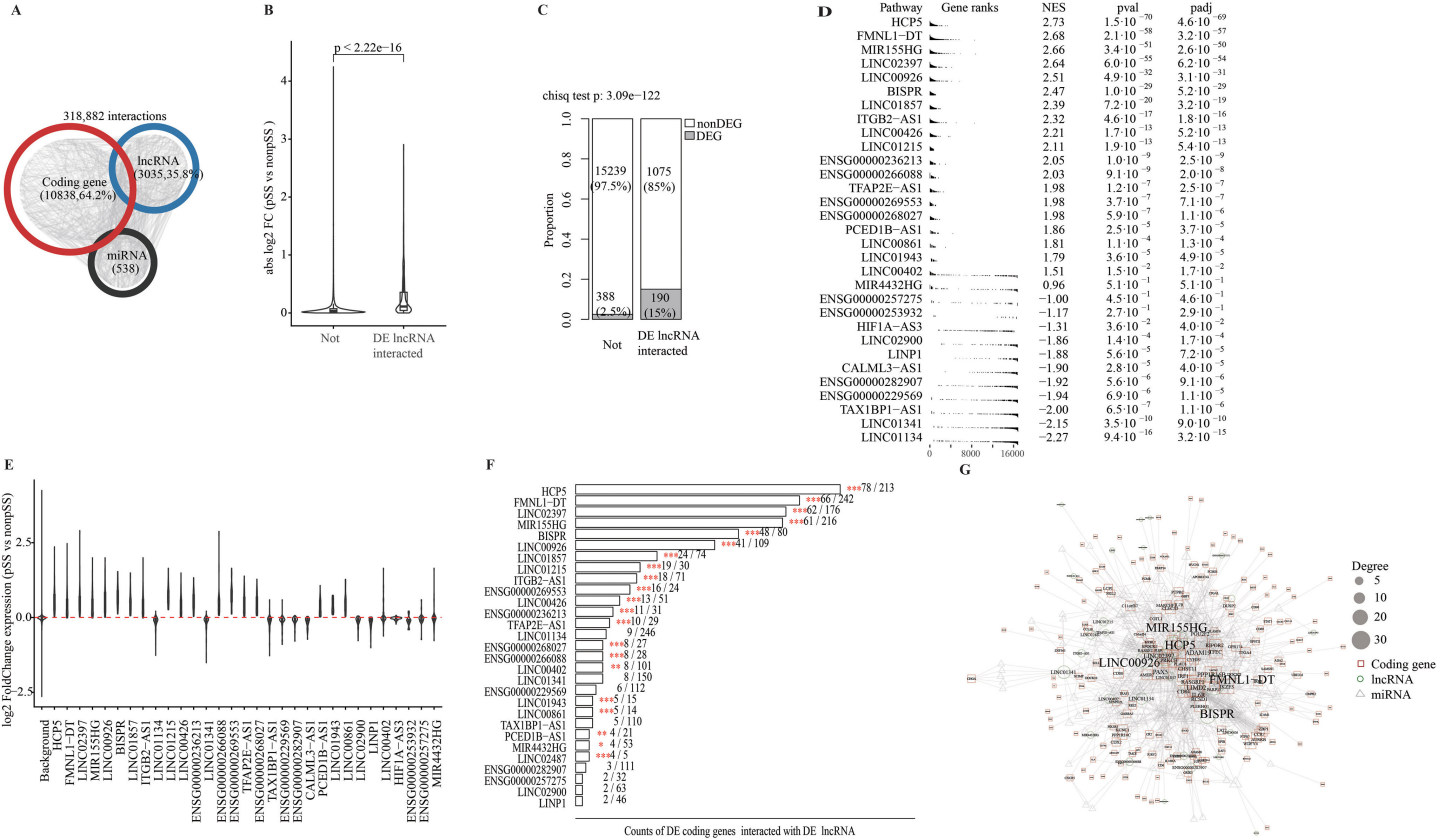
Genome-wide ceRNA network construction and lncRNA regulation function investigation. **(A)** Statistics about coding and lncRNA genes in the ceRNA network constructed in minor salivary gland samples. **(B)** Comparison of expression changes in pSS vs. non-SS for coding genes interacting with pSS DE lncRNAs or not. The absolute log2-transformed gene expression fold change between pSS and non-SS is shown on the *y*-axis. The Wilcoxon rank test was used for comparison of expression changes between coding genes interacting with pSS DE lncRNAs or not, and the resultant *p*-value was labeled on top. **(C)** Proportion of pSS DE coding genes interacting with pSS DE lncRNAs or not. The counts of DE coding gene in each group were shown as stacked bar plot with proportions labeled. The chi-square test was used for testing the DE coding gene proportion difference between interacting with pSS DE lncRNAs and not, and the *p*-value is labeled on top. **(D)** pSS vs. non-SS expression differences for coding genes interacting with pSS DE lncRNAs. Gene ranks, NES, *p*-value, and *p*_adj_ from GSEA are shown for each lncRNA. GSEA was used to test the significance of expression differences between pSS and non-SS for coding genes interacting with each pSS DE lncRNAs (pathway). **(E)** Distribution of gene expression changes between pSS and non-SS for coding genes interacting with each pSS DE lncRNA. The *y*-axis is for log2-transformed gene expression fold changes and the *x*-axis is for each lncRNA. The first column for all expressed coding genes as background. The red dashed line labels fold change of 0. **(F)** Counts of pSS DE coding genes interacting with each pSS DE lncRNA. The red star indicates significantly higher proportion as tested by hypergeometric test: *p < 0.05, **p < 0.01, and ***p < 0.001. **(G)** Interaction relationship between pSS DE lncRNAs, miRNAs, and coding genes on the ceRNA network. Different shapes and colors for biotypes and dot size for number of interaction partners (degree).

The above analyses of lncRNA–coding gene expression correlations and differential expression indicate the potential regulation of coding genes by lncRNAs. The predominance of positive correlations between lncRNA and coding genes is also consistent with ceRNA regulation mechanisms. In addition, it was found that lncRNA–coding gene pairs with shared miRNA binding sites (see Methods) tend to show significantly higher positive expression correlations than random gene pairs (**Figure 2F**), which also support the ceRNA regulation effects. Therefore, we are trying to construct ceRNA network for all lncRNAs and mRNAs genome-widely. The total transcriptome sequencing data of more than 100 MSG samples here enabled us to identify gene expression correlation genome-widely. The lncRNA and coding genes with shared miRNA binding sites (predicted and experimentally validated) and significant positive expression correlation (C.C > 0.5, adjusted *p*-value < 0.05) in MSG samples were used to construct a putative lncRNA–mRNA ceRNA interaction network as mostly done (41, 50) (see **Supplementary Methods** for details). Ultimately, a genome-wide ceRNA network comprising 318,882 interactions between 3,035 lncRNAs (35.8% of all expressed lncRNAs) and 10,838 coding genes (64.2% of all expressed coding genes) were obtained (**Figure 3A**). The ceRNA network fit well for a scale-free network for lncRNA and coding genes separately based on the power law tests (**Supplementary Figure 4A**). Among all pSS DE genes, 44 (28%) pSS DE lncRNA genes and 267 (46%) pSS DE coding genes were present in the ceRNA network (**Supplementary Figure 4B**; **Supplementary Table 1D**).

### Identification of key regulatory lncRNAs involved in pSS pathogenesis

To evaluate the regulatory effects of lncRNAs on coding gene expression changes in pSS as ceRNA, the expression fold change between pSS and non-SS samples for coding genes interacting with ≥1 pSS DE lncRNAs on the ceRNA network was compared with those non-interacting lncRNA genes. As a result, significantly large expression changes were observed for coding genes interacting with ≥1 DE lncRNAs than others (**Figure 3B**). Consistently, among coding genes interacting with any DE lncRNAs, 15% show significant expression changes in pSS vs. non-SS, which is significantly higher than 2.5% for non-interacting genes (**Figure 3C**). To identify specific regulatory lncRNA, the GSEA was utilized to test whether the interactors of each of the 31 pSS DE lncRNAs showed coordinated expression changes in pSS versus non-SS. As a result, the GSEA identified 28 lncRNAs whose interactors showed significantly large expression changes in pSS (adjusted *p* < 0.01; **Figure 3D**), with HCP5, FMNL1-DT, BISPR, LINC02397, and LINC00926 among the top ones. It is of note that expression of interacting coding genes was found to be changed in the same direction (pSS vs. non-SS) as their corresponding lncRNAs (**Figures 3D,E**). Analysis of enrichment of pSS DE coding genes among interacting genes for each lncRNA found significant enrichment for 21 lncRNAs (adjusted test *p*-value < 0.001, **Figure 3F**). A subnetwork of the ceRNA network consisting of the pSS DE lncRNAs, their interacting coding genes, and mediated miRNAs is shown in **Figure 3G**, and lncRNAs such as HCP5, BISPR, LINC00926, and MIR155HG are highly connected. Therefore, analyses of the expression changes for interacting coding genes in pSS underscore the regulatory roles of 21 lncRNAs in biogenesis of the disease. Function enrichment analysis for interacting coding genes of the lncRNAs have found significant enrichment for some infection-related and immune-system processes (**Supplementary Figure 4C**).

### pSS-related pathway activities regulated by lncRNAs

**Figure 4:**
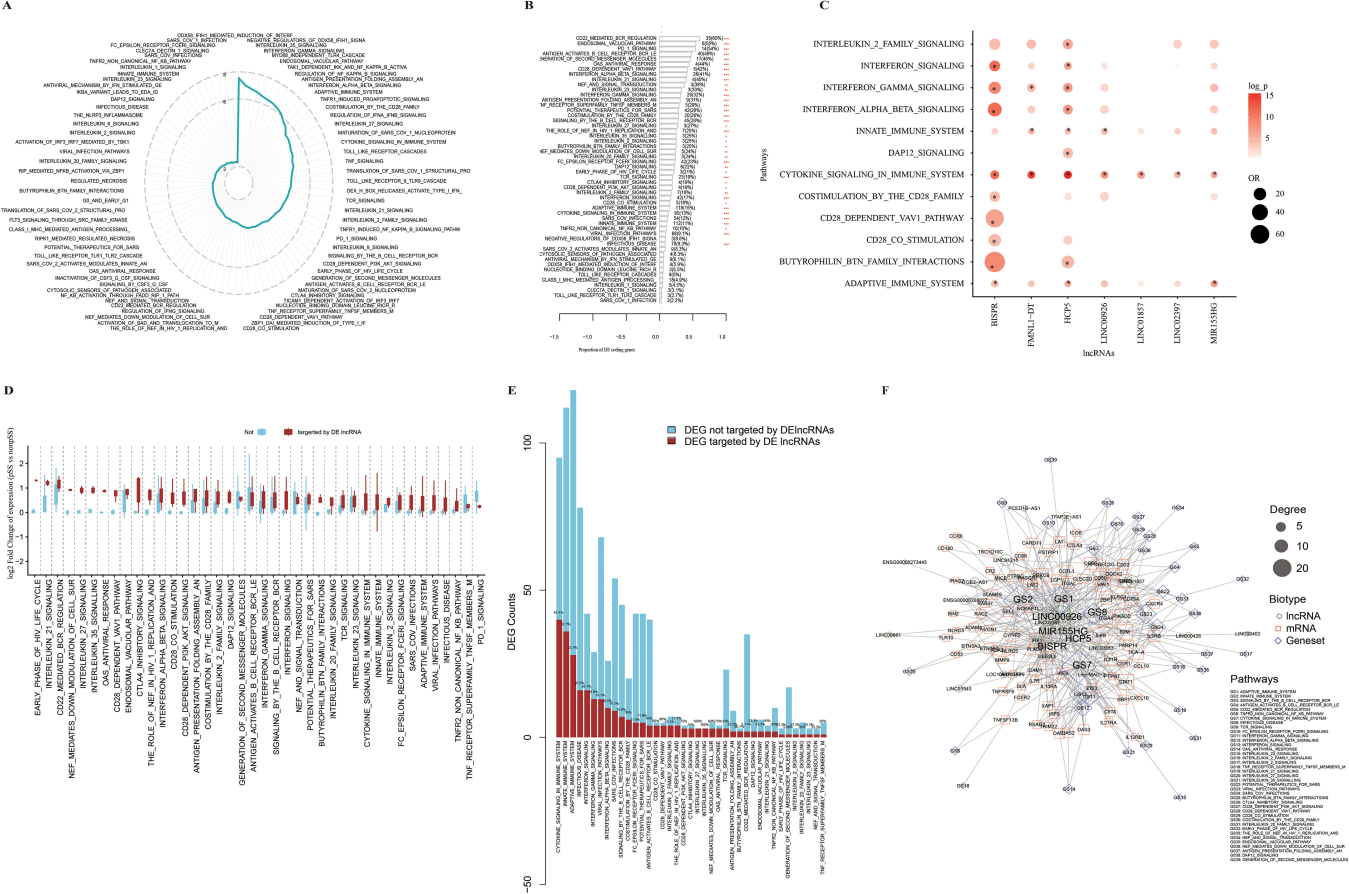
pSS-associated pathway expression regulation by lncRNAs. **(A)** Radar plot of absolute log-adjusted *p*-values for biological pathways significantly associated with pSS. Summed expression level for each pathway in each sample was calculated using the GSVA and pathway expression difference between pSS and non-SS samples was tested using Wilcoxon rank test. Out of 1,692 Reactome pathways showing significant expression changes in pSS vs. non-SS (FDR < 0.05), 82 are presented on the radar plot. **(B)** Proportion of pSS DE coding genes in each of the 40 pathways with ≥3 DE coding genes. Enrichment of pSS DE coding genes in each pathway was tested using the hypergeometric test. The red star labels pathways with significant enrichment: * for FDR < 0.05, ** for FDR < 0.01, and *** for FDR < 0.001. **(C)** Enrichment of interacted coding genes within the pathways for each pSS DE lncRNA. For each pathway, the counts of member genes interacting with each lncRNA on ceRNA network was obtained. The *x*-axis is for lncRNAs, and the *y*-axis is for pathways. Enrichment of lncRNA interacting genes in each pathway compared with all expressed genes was tested using the hypergeometric test. Dot size for odds ratio of gene enrichment and color density for −log10-transformed adjusted *p*-value. lncRNAs showing significant enrichment in the pathway (adjusted *p*-value < 0.05) were labeled using *. **(D)** Gene expression changes in pSS vs. non-SS for coding genes interacting with any DE lncRNAs or not. The *y*-axis is for log2-transformed fold change of expression in pSS vs. non-SS for each gene, and the *x*-axis is for each pathway. Boxplot to represent log2-transformed fold change of expression distribution, with the red box for DE lncRNA interacting genes, and the blue box for non-interacting genes. **(E)** Counts of pSS DE coding genes interacting with any DE lncRNAs in each pathway. The stacked bar plot shows counts of pSS DE coding genes interacting with any pSS DE lncRNA (red) or not (blue). Proportion of member genes interacting with pSS DE lncRNAs in each pathway were labeled on bar top. **(F)** Interaction network between pSS DE lncRNAs, coding genes, and pathways. Different shapes and colors for different entities of lncRNA, coding gene, and pathway, respectively. Dot size for degree.

Many biological processes were frequently reported to be dysregulated in pSS, including IFN signaling and antigen processing and presentation (2, 51, 52), and it is interesting to estimate the contribution of lncRNAs to these changes. Firstly, activity for 1,692 REACTOME pathways were measured using GSVA and compared between pSS and non-SS samples, which resulted in 82 significantly differentially expressed pathways (Wilcoxon test, adjusted *p*-value < 0.05, **Figure 4A**; **Supplementary Table 4**, **Supplementary Methods**). Enrichment analysis of pSS DE genes within the 82 pathways has found 40 pathways showing significant enrichment (pSS-associated pathways, **Figure 4B** hypergeometric test, adjusted *p*-value < 0.05). Notably, most of the pathways are autoimmune diseases-related like BCR activation, IFN signaling, and cytokine signaling. Enrichment of interacting genes of lncRNAs within each of the 40 pathways was tested and 12 pathways showing significant enrichment (hypergeometric test adjusted *p* < 0.05) for seven pSS DE lncRNAs were found (**Figure 4C**). It is of note that in 30 of the 40 pSS-associated pathways, member genes interacting with any DE lncRNAs show much larger expression changes (pSS vs. non-SS) than non-interacting member genes (**Figure 4D**). Furthermore, in 30 of 39 tested pSS-associated pathways, more than 20% of pSS DE coding genes were interactors of lncRNAs on the ceRNA network (**Figure 4E**). Visualization of the subnetwork for pSS DE lncRNAs, their interacting coding genes, and pSS-associated pathways has revealed several lncRNAs like HCP5, BISPR, MIR155HG, and LINC00926 as hub (**Figure 4F**). Moreover, immune function genes like STAT, CD80, and PRKCB were found to interact with many pSS DE lncRNAs.

The results from above indicate that lncRNAs may contribute significantly to pSS-associated pathway expression changes via ceRNA mechanisms.

### Validation of the regulatory effect of key lncRNAs as ceRNA in A253 cell lines

**Figure 5:**
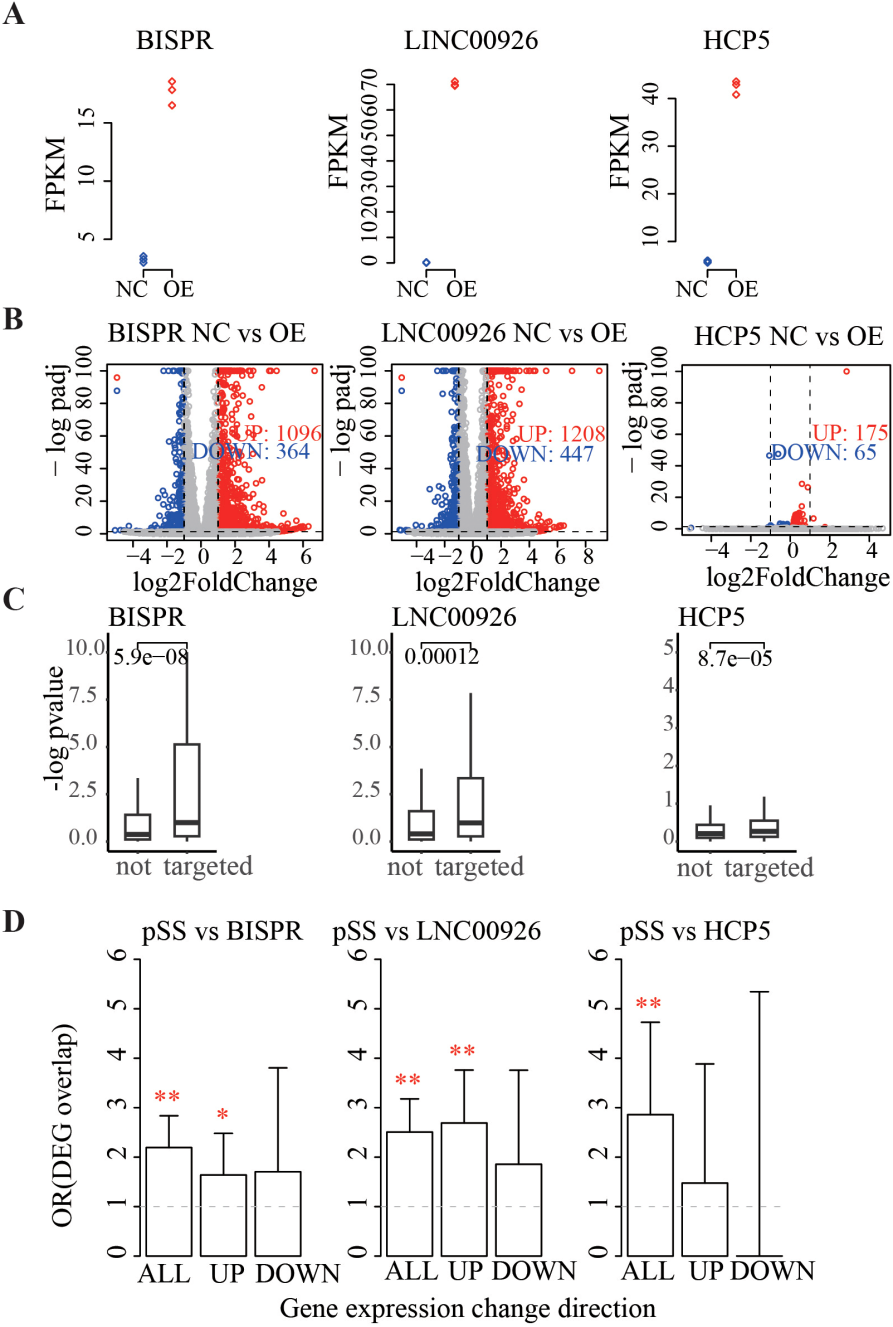
Validation of regulatory effect for pSS DE lncRNAs using overexpression experiment in A253 cell lines.**(A)** Expression level for BISPR (left), LINC00926 (center), and HCP5 (right) in three overexpression (OE) cell lines and three control cell lines (NC). Red diamond for OE and blue for NC. The *y*-axis is for expression level estimated using FPKM from RNA-seq data. **(B)** The volcano plot shows gene expression differences in NC vs. OE cell lines. Expression level fold change (OE/NC, *x*-axis) and adjusted *p*-values (*y*-axis) are shown. Dashed lines for a fold change of 2 and an adjusted *p*-value of 0.05. Red denotes significant differentially expressed genes and gray indicates non-significant genes. OE cell lines for BISPR (left), LINC00926 (center), and HCP5 (right) are shown. **(C)** Box plot shows significance of gene expression differences between lncRNA OE and NC cell lines. The *y*-axis is for −log10-transformed *p*-values from gene expression difference test between OE and NC cell lines. The expression difference test *p*-values for genes interacting with lncRNA or not were compared using Wilcoxon test, and the *p*-value is labeled on top. Left for BISPR, center for LINC00926, and right for HCP5. **(D)** Overlap between differentially expressed genes detected in pSS vs. non-SS samples and in lncRNA OE vs. NC cell lines. Bar height shows odds ratio (OR) of differentially expressed gene (DEG) overlap between the two datasets with error bars to show 95% confidence interval. Genes with different change directions are shown separately: ALL denotes all DEGs, UP indicates upregulated DEGs in both pSS/non-SS and OE/NC, and DOWN represents downregulated DEGs in both pSS/non-SS and OE/NC. Fisher’s exact test was used for test of significance of DEG overlap, with asterisks denoting significance labeled on top: *p < 0.05 and **p < 0.01. The dashed line labels overlap OR of 1 (no significance).

Three pSS-associated lncRNAs—BISPR, LINC00926 and HCP5—supposed to be key regulatory ceRNAs based on the above analysis (**Figures 3D,F**, **4C**) were picked to validate their regulatory effects using overexpression experiments in cell lines. The human salivary gland epithelial cell lines A253 was used here since its function is related to pSS biogenesis (53). The three lncRNAs were OE via lentiviral infection in A253 cells (>90% efficiency, **Supplementary Figure 5A**) to produce three biological replicates for each lncRNA. In addition, three empty-vector infected A253 cell replicates were produced as NC (see Methods). Standard transcriptome sequencing (RNA-seq) was performed on each replicate to obtain expression for genome-wide genes (see Methods). Firstly, expression for the three lncRNAs was elevated more than five times in the OE cell line vs. control (**Figure 5A**), which confirms successful transfection. DEGs between three OE replicates and three NC replicates (OE–NC DEGs) were detected for each lncRNA overexpression experiment using the DEseq2 (adjusted *p*-value < 0.05 and absolute fold change >2). As a result, 1,460, 1,655, and 3 DEGs were detected for BISPR, LINC00926, and HCP5, respectively (**Figure 5B**). The few DEGs detected in HCP5 (3) indicate its week regulatory effect. Then, the DEG calling criteria for HCP5 overexpression experiment were relaxed to adjusted *p*-value < 0.3, with 240 DEGs detected for downstream analysis. Notably, significantly more upregulated than downregulated genes were detected in lncRNA OE cell lines compared to NC (3, 2.7, and 2.69 times more for BISPR, LINC00926, and HCP5, respectively), which is expected given the positive expression correlation between ceRNA interactors.

To validate lncRNA’s regulatory roles and the reliability of the genome-wide ceRNA network constructed from MSG transcriptomes above, we tested whether genes interacting with lncRNAs respond to lncRNA perturbation (overexpression). For each of the three lncRNAs, OE–NC expression differences were compared between interacting and non-interacting genes (on the ceRNA network constructed above), and the former showed substantially larger expression changes (**Figure 5C**). Consistent with this, the proportion of OE–NC DEGs detected among interacting genes were much higher than among non-interacting genes for each of the three lncRNAs: 11.2% vs. 4.2% for BISPR (*p*-value: 0.0009), 6.7% vs. 4.8% for LINC00926 (*p*-value: 0.288), and 1.7% vs. 0.7% for HCP5 (*p*-value: 0.04) (**Supplementary Figure 5B**). These findings further support gene expression regulation roles for the three lncRNAs as ceRNA.

To directly estimate the regulatory effects of lncRNAs on gene expression changes in pSS, DEGs detected in pSS vs. non-SS samples were compared with those in lncRNA OE vs. NC cell lines. As a result, a significantly higher proportion of pSS vs. non-SS DEGs were also found to be lncRNA OE vs. NC DEGs: 11.9% (71/597, OR: 2.29) for BISPR, 14.8% (87/589, OR: 2.5) for LINC00926, and 2.85% (17/593, OR: 3.88) for HCP5 (**Figure 5D**, ALL bar). When change directions are considered, a significant overlap for upregulated DEG genes between the two datasets was found (OR: 1.64, 2.69, and 1.47 for BISPR, LINC00926, and HCP5, respectively) (**Figure 5D**, UP bar), whereas overlap for downregulated genes was not significant (**Figure 5D**, DOWN bar). In addition, GSEA revealed that pSS-associated immune processes were significantly altered in the lncRNA OE cell lines, with representative genes such as IFI44L, IL2RB, XAF1, and GBP1 showing concordant changes (**Supplementary Figures 5C,D,E,F**).

### Association between lncRNA expression and clinical characteristics of pSS

**Figure 6:**
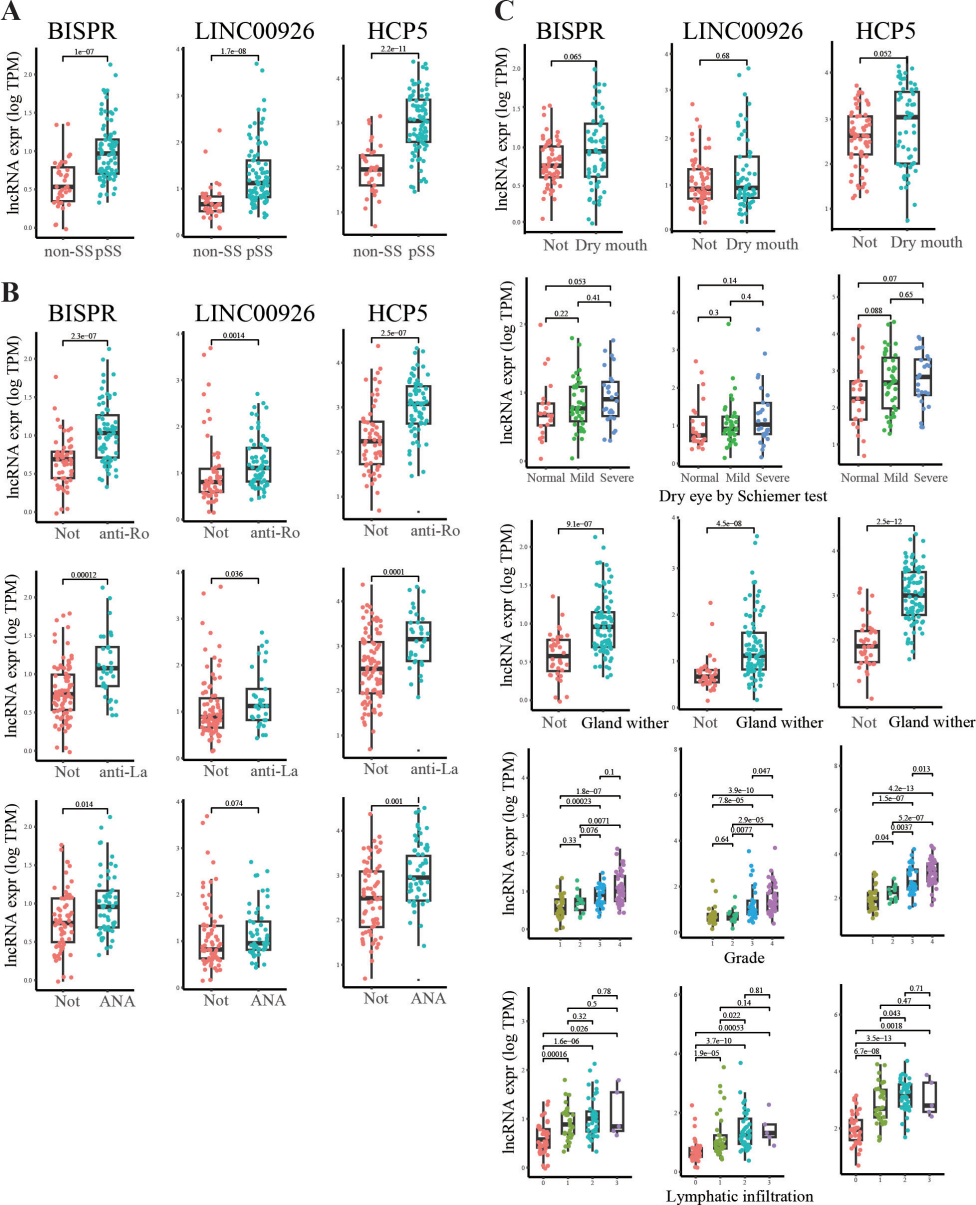
Relationship between presence of clinical features and the three key pSS DE lncRNAs’ expression level. **(A)** Expression level for BISPR (left), LINC00926 (center), and HCP5 (right) in non-SS and pSS samples. Each dot for one sample; the y-axis is for log-transformed TPM and the Wilcoxon test was used for comparison of expression, with *p*-value labeled above. **(B, C)** Similar to **(A)** but for samples from individuals with different autoantibody presence **(B)** and disease severity-related clinical features **(C)** as labeled on the *x*-axis. Not means no autoantibody detected or no symptoms present.

While the three lncRNAs—BISPR, LINC00926, and HCP5—were identified as candidate regulators of gene expression changes in pSS based on the above analysis, it would be meaningful to investigate their relationship with clinical features of pSS. Firstly, the expression levels for the lncRNAs were significantly higher in pSS than in non-SS (**Figure 6A**). The three key lncRNA genes were also found to be significantly upregulated in individuals with the presence of autoimmune autoantibodies: anti-Ro/SSA, anti-La/SSB, and anti-nuclear (ANA) (**Figure 6B**). In addition, the lncRNA expression was also found to be correlated with measures of disease severity such as grade, dry eye/mouth symptoms, and gland withering (**Figure 6C**).

## Discussion

Many studies have been performed to analyze gene expression changes in patients with pSS compared with healthy or non-SS individuals (54, 55), which helps with the understanding, diagnosis, and subtyping of the disease. Most studies of gene expression in pSS focus on coding genes (56, 57) considering their well-annotated functions, but the mechanisms underlying gene expression changes are unclear and rarely studied. Although lncRNA expression in pSS has been examined and many dysregulated lncRNAs have been reported, their functions largely remain uncertain (20, 25). In fact, lncRNA could regulate gene expression in various manners with ceRNA interaction as one common way (39, 58–61). Moreover, lncRNA-mediated ceRNA regulation has been extensively studied in cancer (31, 50, 62, 63), but has been scarcely investigated in pSS. In this work, we have, for the first time, systematically investigated the regulatory role of lncRNA in driving coding gene expression changes in pSS. The deep total transcriptome sequencing of MSG samples from 126 patients with pSS and non-SS subjects enables genome-wide analysis of both coding and lncRNA gene expression. To our knowledge, this is the largest transcriptome-sequencing dataset of the primary affected tissue in pSS to date.

Analysis of the genome-wide distribution of pSS DE genes reveals non-uniform patterns (**Figure 1C**) and enrichment around genetic association locus reported by GWAS like HLA regions (1, 48) (**Figures 1E,F**). These findings indicate that pSS-associated gene expression changes could be regulated by nearby genetic elements (in cis). The lncRNA genes and coding genes located nearby tend to change concordantly between pSS and non-SS samples, which also indicates their regulatory interactions (**Figures 2A,B**). However, only a small proportion of pSS DE genes could be regulated in cis based on the above analysis (**Figure 1D**, Additional file 4: **Supplementary Figure 3c**; **Supplementary Table 3**). Combined studies of the lncRNA–coding gene co-expression relationship and their expression changes in pSS support the widespread regulatory roles of lncRNA in pSS pathogenesis (**Figures 2C,D,E**). LncRNA–coding gene pairs with shared miRNA binding sites tend to show higher expression correlation (**Figure 2F**), which indicates ceRNA interactions. Accordingly, we constructed a genome-wide ceRNA network in MSG samples comprising 2,046 lncRNAs and 11,061 coding genes (**Figure 3A**). One large ceRNA network was constructed across 12 cancers, which contain 252 lncRNAs and 1,176 mRNAs (39). Thus, to our knowledge, ours is the largest and most comprehensive ceRNA network.

In this work, we have proven the validity of the genome-wide ceRNA network by showing that coding genes and interacted lncRNAs tend to change concordantly in pSS vs. non-SS (**Figures 3B,C**). We have identified 21 lncRNAs whose interacting genes on the ceRNA network are significantly enriched for pSS DE genes (**Figures 3D–F**), which indicates the critical regulatory role of these lncRNAs in pSS pathogenesis. Notably, 5 of the 21 lncRNAs were reported to function as ceRNAs: HCP5 (60, 64–66), linc00926 (58, 67), Linc00426 (59, 68), linc01215 (62), and Linc01857 (69, 70). BISPR was also reported to be a key regulator of coding genes’ expression in pSS (21). Previous studies have found many dysregulated biological pathways in pSS, but the underlying mechanisms are unknown. In our work, we have found that activity for many pSS-related pathways could be regulated by few lncRNAs based on the significant enrichment of interacting genes in each pathway (**Figure 4C**) and their larger expression changes in pSS vs. non-SS (**Figure 4D**). Regulatory effects for three critical regulatory lncRNAs were validated in human salivary gland-derived cell lines (**Figure 5**), which support the reliability of our ceRNA network.

In this work, the largest study of MSG by total transcriptome sequencing has identified more than 200 lncRNA genes differentially expressed between pSS and non-SS (**Supplementary Figure 2C**), some of which could be used as diagnosis biomarkers. Systematic analysis of pathway expression has recapitulated the previous reported upregulation of IFN-pathway genes in pSS. The enrichment of lncRNA-interacting genes indicates the possible contribution of lncRNA to these pathway expression changes via ceRNA mechanisms. Therefore, the manipulation of lncRNA expression could possibly be used as treatment of pSS. For example, knockdown of BISPR could reduce human microglial inflammation (71); HCP5 gene knockdown could suppress the inflammation and oxidative stress in rat model (72). LINC00926 is shown to regulate the expression of WNT10B and consequently inflammation in post-traumatic stress disorder (PTSD) (73).

We acknowledge that the lncRNA–mRNA ceRNA network constructed based on gene expression correlation and predicted miRNA binding sites from other sources may contain some false positives. Nevertheless, our genome-wide analyses indicate widespread lncRNA involvement consistent with ceRNA activity in pSS and recommend high-confidence regulator candidates that merit further study. The confident candidates related to the disease could be further validated using miRNA-seq and CLIP experiment from the same samples. We note that immune cell infiltration is a hallmark of MSG in pSS and can confound gene expression correlations derived from bulk-tissue sequencing as used here. To mitigate this, we used *in silico* deconvolution (CIBERSORTx) to estimate immune-cell proportions per sample and adjusted expression values prior to correlation and network construction. Our experiment validation of the lncRNA regulation effect on cell lines support their function as ceRNA inferred from the transcriptome sequencing data analysis of MSG samples. Taken together, these adjustments and experimental validations indicate that immune-infiltration effects are unlikely to undermine the main conclusions. We could expect that single-cell sequencing would provide a superior solution to the cell mixture problem in bulk tissue.

Our study demonstrates that lncRNAs make significant contributions to gene expression changes in MSGs of patients with pSS. The comprehensive ceRNA network constructed could be used for lncRNA function inference based on their interacting coding genes. We have identified and experimentally validated several key lncRNAs with strong regulatory effects on coding genes, and their associations with clinical features of pSS support an important role in disease pathogenesis.

## Data Availability

The data presented in the study are deposited in the Genome Sequence Archive for human repository (https://ngdc.cncb.ac.cn/gsa-human/), accession number: HRA017750.

## Supplementary material

Supplementary Figure 1 Validation of novel lncRNA detected from total transcriptome sequencing data. **(A)** Reads coverage and transcript structure for 2 novel lncRNAs assembled using total transcriptome sequencing data on one MSG sample as example. Left for MSTRG.25070 and right for MSTRG.45513. The plot produced from IGV software.**(B)** expression level for the 2 novel lncRNAs based on total transcriptome sequencing data for pSS (red) and non-SS (blue) samples. Y-axis for expression estimated as TPM and Wilcoxon test p value for expression differences between pSS and non-SS are labeled on top. Left for MSTRG.25070 and right for MSTRG.45513. **(C)** Northern Blotting results for PCR amplification products of MSTRG25070 (lane 1,2,3) and MSTRG45513 (lane 4,5,6) using specific primers in three MSG samples. M for marker. The PCR product size for MSTRG25070 is 144 nt and it is 127 nt for MSTRG45513 as labeled on image plot using red arrow. (see Methods) **(D)** Chromatogram and nucleotide sequences determined for PCR products of MSTRG.25070 (upper) and MSTRG45513 (lower) using sanger sequencing. **(E)** Alignment of nucleotide sequence for MSTRG.25070 (upper) and MSTRG45513 (lower) to their original location on human genome (hg38). The nucleotide sequences were determined by sanger sequencing of PCR products of the two novel lncRNAs. Their original location was determined by read alignment of sequencing reads. The alignment visualization is produced by UCSC genome browser. See methods for details.

Supplementary Figure 2 Differentially expressed genes detection between pSS and non-SS samples and functional enrichment analysis. **(A)** Distribution of expression level (log TPM) and coefficient of variation (CV) for protein coding, known lncRNA and novel lncRNA genes separately. **(B)** Principal Component Analysis of samples using expression level for all expressed genes. Gene expression level is the log2 transformed TPM and quantile normalized. X-axis for the first component and y-axis for the second. each dot for one sample and color for different groups: pSS (black) and non-SS (grey). **(C)** Counts and proportion of up-regulated or down-regulated DEGs for novel lncRNA, known lncRNA and coding genes in pSS compared with non-SS samples. Gene counts and proportion among all expressed genes are labeled on each bar and different colors for changing direction. **(D)** Overlap between pSS DE genes detected in our study cohort and those reported before by other publications, for coding genes (left) and lncRNA genes (right). Here the blue for published DEGs in pSS, red for detected in our data, and common for overlap. The p value and odds ratio from hypergeometric test of overlap are labeled. **(E)** GO BP term enrichment for DE coding genes up-regulated in pSS. Y-axis for GO BP terms and x-axis for gene ratio, with dot size for counts of DEGs in each term and color density for adjusted p value. Top ten enriched GO BP terms are shown. The function enrichment analysis is performed using Clusterprofile software. **(F)** Similar as **(E)**, but for pSS down-regulated DE coding genes in pSS compared with non-SS samples.

Supplementary Figure 3 Genome-wide distribution and correlation analysis of pSS DEGs. **(A)** Overview of pSS DEGs across all genome regions. Dot with different color and shape for DEG of different biotypes and numbers for chromosomes. **(B)** Enrichment of pSS DEGs in each chromosome. Enrichment of DEG was test by hypergeometric test using whole genome expressed genes as background. The x-axis for odds ratio of enrichment, and vertical dashed line for odds ratio of 1. Enrichment significance was labeled: * for p value < 0.05, ** for p value < 0.01. **(C)** Visualization of distribution of genes for two pSS DEG clusters detected on chr6. Dark red for DEGs and pink for non-DEGs, square for coding genes and dot for lncRNA genes. The gene names are labeled. The DEG clusters was detected based on maximum nearby DEG gene distances < 100 kbp (based on TSS) and with minimum of 3 genes. (see Methods for details). X-axis for genome location (hg38) and y-axis for log2 fold change of expression in pSS vs non-SS. Upper and lower for two different pSS DEG clusters. **(D)** Enrichment of significant expression changes in pSS vs non-SS on chromosome cytoband using GSEA. The average expression is calculated for pSS and non-SS samples. The 4 cytoband regions with significant enrichment of gene expression changes are shown (adjusted p value < 0.1). **(E)** Proportion of pSS DEGs among reported disease association genes and all other genes. X-axis for two gene groups: 697 reported disease association genes collected from published GWAS studies and all other expressed genes detected in our dataset. Y-axis for proportion of pSS DEG among each group of genes. p value and odds ratio from hypergeometric test of proportion differences between the two gene groups were labeled on top. **(F)** Proportion of genes with different number of correlated lncRNAs among pSS DE and non-DE coding gene. Coding genes are broadly divided into 3 groups based on number of correlated lncRNA genes: 0 (NONE), 1-10(FEW) and >10(MANY). Proportion of genes with different number of correlated lncRNA gene counts for DE and non-DE coding genes were shown as stacked bar plot and compared by the Chi-square test, with p value shown. Here expression correlation between genes was calculated in the human MSG samples from Gtex database using the Pearson correlation test, see Methods for details. **(G)** Co-expression gene clusters identified by WGCNA software tool. Hierarchy clustering of 20875 genes based on co-expression result in 58 co-expression modules.

Supplementary Figure 4 lncRNA function analysis in ceRNA network constructed. **(A)** Power law test for genome-wide ceRNA network for coding genes (left) and lncRNA genes(right) respectively. X-axis for network degree, and y for distribution. The red line for fit line. The higher power law test p value means no significant differences from network following power law. **(B)** Counts and proportion of pSS DEGs in the genome-wide ceRNA network contracted. Left for lncRNA and right for coding genes. **(C)** Function enrichment analysis for interacting coding genes for each pSS DE lncRNA. The KEGG pathway enrichment was performed and top 20 enriched terms were shown. The dot size for counts of genes in each pathway and color density for enrichment significance as tested by hypergeometric test. pSS DE lncRNAs with significant function enrichment were shown here.

Supplementary Figure 5 lncRNA regulation effect validation using overexpression experiment in A253 cell lines. **(A)** Image of infection efficiency of the lentiviral plasmids expressing human lncRNAs into A253 cells. The 3 lncRNAs: BISPR (left), LINC00926 (middle) and HCP5 (right) were shown. **(B)** Proportion OE vs NC DEGs among genes interacting with lncRNA (Reg) or not (Not) in OE cell line vs NC. The OE cell lines produced for BISPR, LINC00926 and HCP5 were shown respectively. The DEG here means differentially expressed genes detected between OE and NC cell lines using RNA-seq data and the Reg means genes interacting with lncRNA based on ceRNA network constructed above in MSG samples. Not for genes not interacting with lncRNAs on ceRNA network. Y-axis for DEG proportions and error bar for 95% confidence interval. The hypergeometric test was used to compare DEG proportion differences among genes targeted or not by lncRNAs on the ceRNA network, with odds ratio and p value shown on top. **(C)** changes of expression of biological pathways between OE cell line and NC cell line by GSEA. X-axis for ordered ranks for genes (upper regulation in OE on left to down regulation in OE on right), and y-axis for enrichment score produced by GSEA. Top significant pathways were presented for each of 3 lncRNAs: BISPR, LINC00926 and HCP5. INTER_S, INTERFERON_SIGNALING; INTER_G_S, INTERFERON_GAMMA_SIGNALING; INTER_AB_S, INTERFERON_ALPHA_BETA_SIGNALING; DAP12_I, DAP12_INTERACTIONS; SIGLA_R, SIGNAL_REGULATORY_PROTEIN_FAMILY_INTERACTIONS; PHOTO_CAS_A, ACTIVATION_OF_THE_PHOTOTRANSDUCTION_CASCADE; PHOTO_CAS, THE_PHOTOTRANSDUCTION_CASCADE; FCGR_A, FCGR_ACTIVATION; INTERL2_S, INTERLEUKIN_2_SIGNALING; INTERL2_F_S, INTERLEUKIN_2_FAMILY_SIGNALING. **(D)** Expression level for 3 auto-immune disease related genes interacting with BISPR. Expression level (FPKM) in 3 lncRNA overexpression cell line (OE) samples and 3 control cell line (NC) samples were shown for each gene. The expression level difference is significant (adjusted p value < 0.05 estimated by DEseq2 software) between 3 OE and 3 NC for each gene shown here. **(E, F)** sample as **(D)** for auto-immune disease related genes interacting with HCP5 and LINC00926 respectively.
