# Supplementary material for: Genome-wide gene expression analysis suggests an important regulatory role of lncRNAs in primary Sjögren’s syndrome
Source: Front Immunol. 2026 Apr 15;17:1751195. doi: 10.3389/fimmu.2026.1751195 (PMC13124706; doi:10.3389/fimmu.2026.1751195)
Supplement: Supplementary Figure 1 — Validation of novel lncRNA detected from total transcriptome sequencing data. (A) Reads coverage and transcript structure for 2 novel lncRNAs assembled using total transcriptome sequencing data on one MSG sample as example. Left for MSTRG.25070 and right for MSTRG.45513. The plot produced from IGV software.(B) expression level for the 2 novel lncRNAs based on total transcriptome sequencing data for pSS (red) and non-SS (blue) samples. Y-axis for expression estimated as TPM and Wilcoxon test p value for expression differences between pSS and non-SS are labeled on top. Left for MSTRG.25070 and right for MSTRG.45513. (C) Northern Blotting results for PCR amplification products of MSTRG25070 (lane 1,2,3) and MSTRG45513 (lane 4,5,6) using specific primers in three MSG samples. M for marker. The PCR product size for MSTRG25070 is 144 nt and it is 127 nt for MSTRG45513 as labeled on image plot using red arrow. (see Methods) (D) Chromatogram and nucleotide sequences determined for PCR products of MSTRG.25070 (upper) and MSTRG45513 (lower) using sanger sequencing. (E) Alignment of nucleotide sequence for MSTRG.25070 (upper) and MSTRG45513 (lower) to their original location on human genome (hg38). The nucleotide sequences were determined by sanger sequencing of PCR products of the two novel lncRNAs. Their original location was determined by read alignment of sequencing reads. The alignment visualization is produced by UCSC genome browser. See methods for details. [file DataSheet1.pdf]

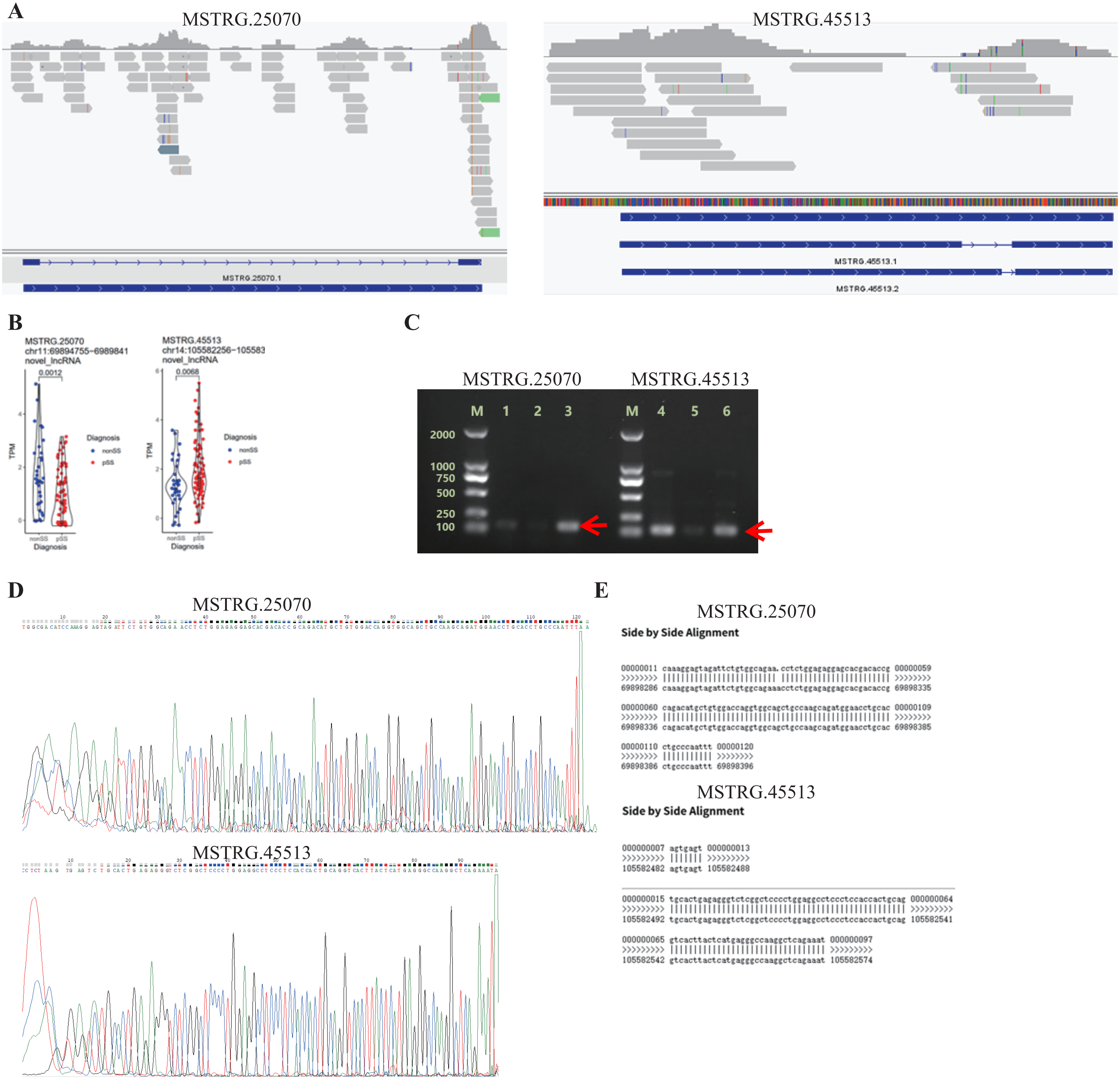
A
MSTRG.25070
MSTRG.25070.1
MSTRG.45513
MSTRG.45513.1
MSTRG.45513.2
B
MSTRG.25070
chr11:69894755-6989841
novel_lncRNA
0.0012
TPM
Diagnosis
nonSS
pSS
MSTRG.45513
chr14:105582256-105583
novel_lncRNA
0.0068
C
MSTRG.25070
MSTRG.45513
M 1 2 3
M 4 5 6
2000
1000
750
500
250
100
D
MSTRG.25070
MSTRG.45513
E
MSTRG.25070
Side by Side Alignment
00000011 caaaggagtagattctgtggcagaa.cctctggagaggagcacgacaccg 00000059
69898286 caaaggagtagattctgtggcagaaacctctggagaggagcacgacaccg 69898335
00000060 cagacatgctgtggaccaggtggcagctgccaagcagatggaacctgcac 00000109
69898336 cagacatgctgtggaccaggtggcagctgccaagcagatggaacctgcac 69898385
00000110 ctgcccaattt 00000120
69898386 ctgcccaattt 69898396
MSTRG.45513
Side by Side Alignment
000000007 agtgagt 000000013
105582482 agtgagt 105582488
000000015 tgcactgagagggtctcggctcccctggaggcctccctccaccactgcag 000000064
105582492 tgcactgagagggtctcggctcccctggaggcctccctccaccactgcag 105582541
000000065 gtcacttactcatgagggccaaggctcagaaat 000000097
105582542 gtcacttactcatgagggccaaggctcagaaat 105582574
