# Supplementary figures and images for: Genome-wide gene expression analysis suggests an important regulatory role of lncRNAs in primary Sjögren’s syndrome
Source: Front Immunol. 2026 Apr 15;17:1751195. doi: 10.3389/fimmu.2026.1751195 (PMC13124706; doi:10.3389/fimmu.2026.1751195)

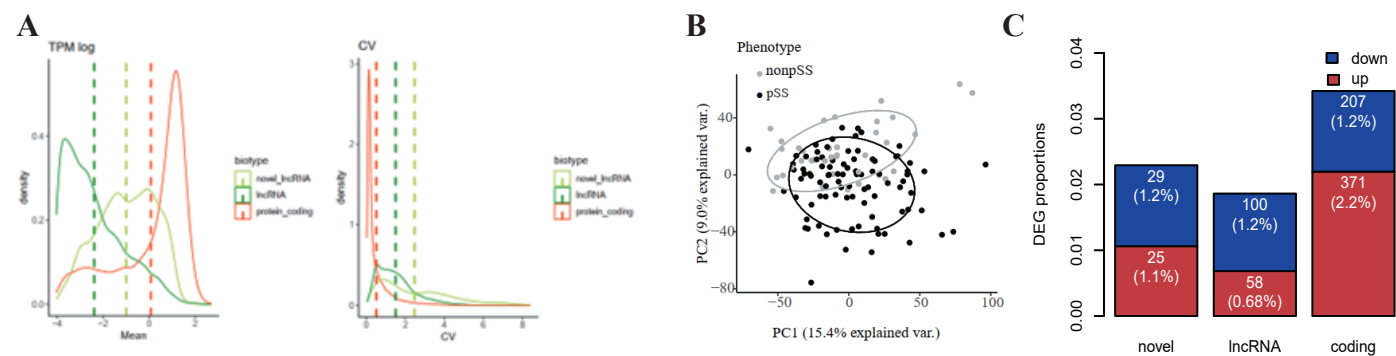

**D**

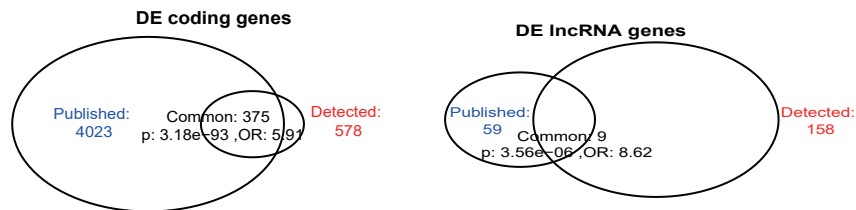

**E**

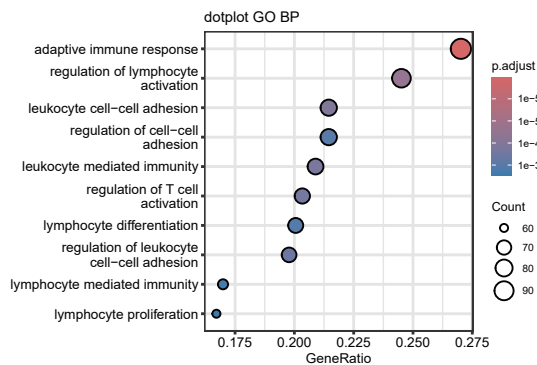

**F**

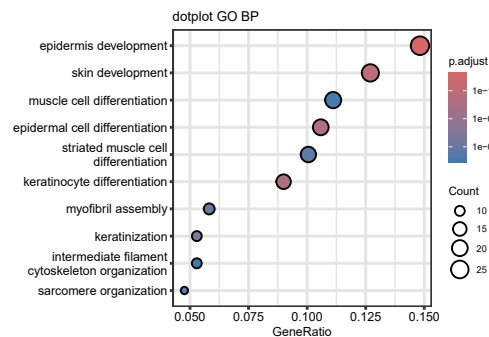

Supplement: Supplementary Figure 2 — Differentially expressed genes detection between pSS and non-SS samples and functional enrichment analysis. (A) Distribution of expression level (log TPM) and coefficient of variation (CV) for protein coding, known lncRNA and novel lncRNA genes separately. (B) Principal Component Analysis of samples using expression level for all expressed genes. Gene expression level is the log2 transformed TPM and quantile normalized. X-axis for the first component and y-axis for the second. each dot for one sample and color for different groups: pSS (black) and non-SS (grey). (C) Counts and proportion of up-regulated or down-regulated DEGs for novel lncRNA, known lncRNA and coding genes in pSS compared with non-SS samples. Gene counts and proportion among all expressed genes are labeled on each bar and different colors for changing direction. (D) Overlap between pSS DE genes detected in our study cohort and those reported before by other publications, for coding genes (left) and lncRNA genes (right). Here the blue for published DEGs in pSS, red for detected in our data, and common for overlap. The p value and odds ratio from hypergeometric test of overlap are labeled. (E) GO BP term enrichment for DE coding genes up-regulated in pSS. Y-axis for GO BP terms and x-axis for gene ratio, with dot size for counts of DEGs in each term and color density for adjusted p value. Top ten enriched GO BP terms are shown. The function enrichment analysis is performed using Clusterprofile software. (F) Similar as (E), but for pSS down-regulated DE coding genes in pSS compared with non-SS samples. [file DataSheet2.pdf]

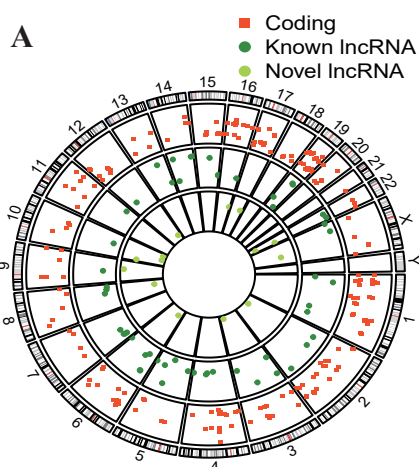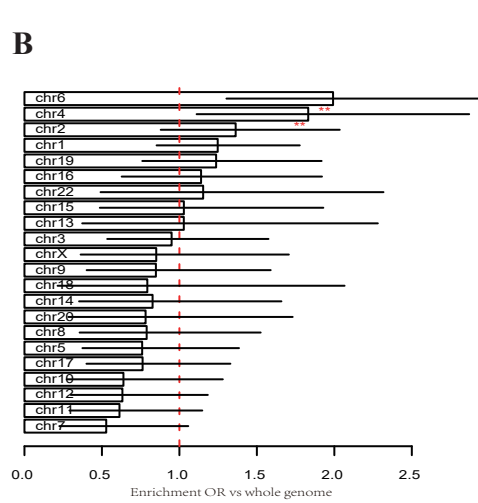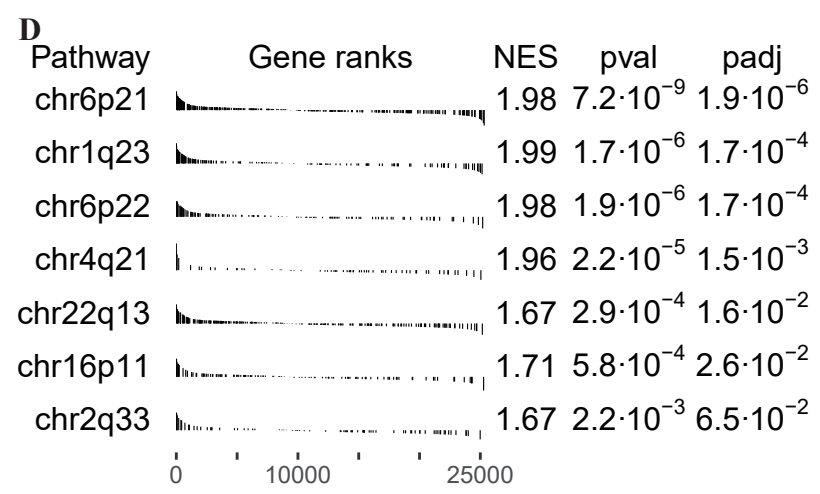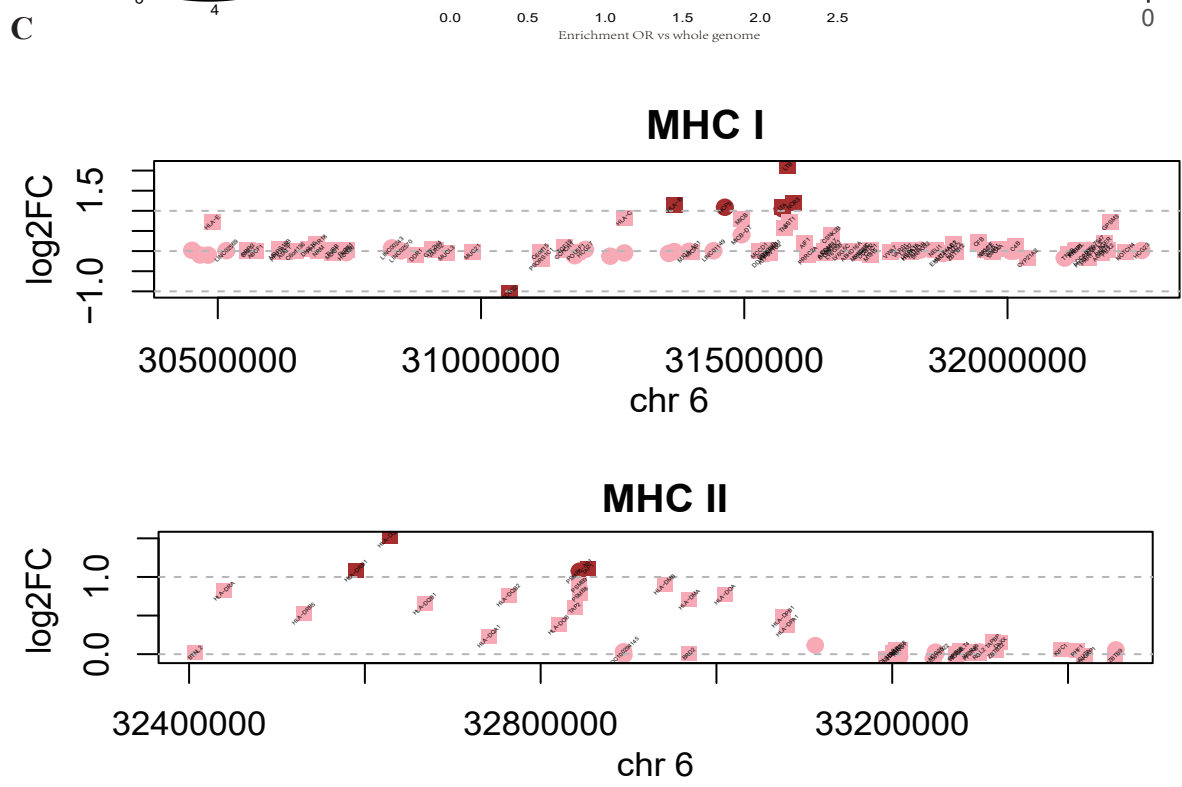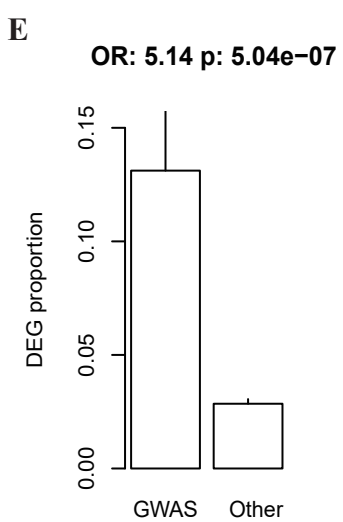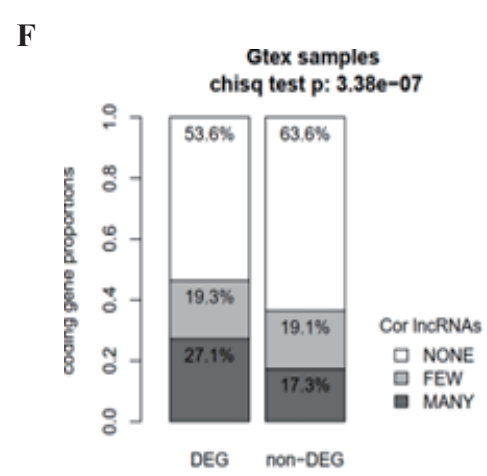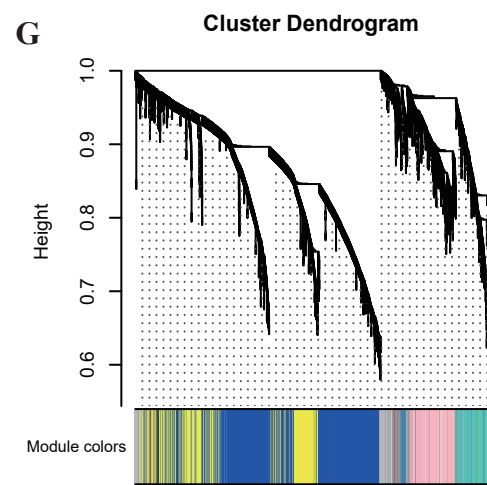

Supplement: Supplementary Figure 3 — Genome-wide distribution and correlation analysis of pSS DEGs. (A) Overview of pSS DEGs across all genome regions. Dot with different color and shape for DEG of different biotypes and numbers for chromosomes. (B) Enrichment of pSS DEGs in each chromosome. Enrichment of DEG was test by hypergeometric test using whole genome expressed genes as background. The x-axis for odds ratio of enrichment, and vertical dashed line for odds ratio of 1. Enrichment significance was labeled: * for p value < 0.05, ** for p value < 0.01. (C) Visualization of distribution of genes for two pSS DEG clusters detected on chr6. Dark red for DEGs and pink for non-DEGs, square for coding genes and dot for lncRNA genes. The gene names are labeled. The DEG clusters was detected based on maximum nearby DEG gene distances < 100 kbp (based on TSS) and with minimum of 3 genes. (see Methods for details). X-axis for genome location (hg38) and y-axis for log2 fold change of expression in pSS vs non-SS. Upper and lower for two different pSS DEG clusters. (D) Enrichment of significant expression changes in pSS vs non-SS on chromosome cytoband using GSEA. The average expression is calculated for pSS and non-SS samples. The 4 cytoband regions with significant enrichment of gene expression changes are shown (adjusted p value < 0.1). (E) Proportion of pSS DEGs among reported disease association genes and all other genes. X-axis for two gene groups: 697 reported disease association genes collected from published GWAS studies and all other expressed genes detected in our dataset. Y-axis for proportion of pSS DEG among each group of genes. p value and odds ratio from hypergeometric test of proportion differences between the two gene groups were labeled on top. (F) Proportion of genes with different number of correlated lncRNAs among pSS DE and non-DE coding gene. Coding genes are broadly divided into 3 groups based on number of correlated lncRNA genes: 0 (NONE), 1-10(FEW) and >10(MANY). Proportion of genes wit [file DataSheet3.pdf]

Power law test p:  
0.89

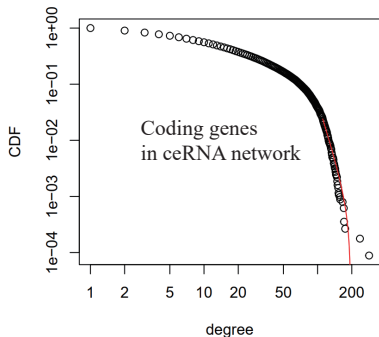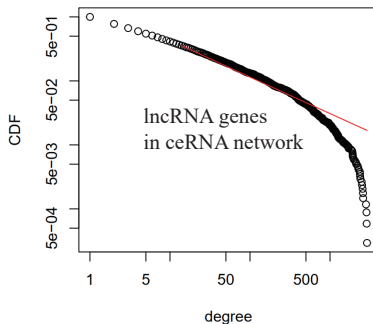

# B

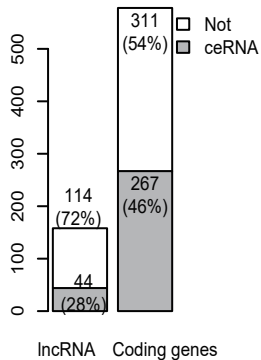

# C

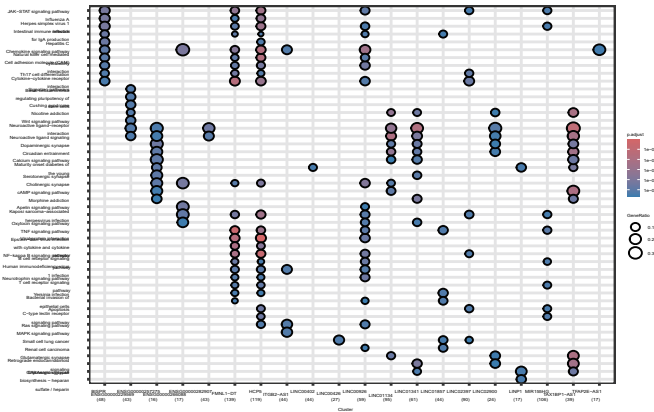

Supplement: Supplementary Figure 4 — lncRNA function analysis in ceRNA network constructed. (A) Power law test for genome-wide ceRNA network for coding genes (left) and lncRNA genes(right) respectively. X-axis for network degree, and y for distribution. The red line for fit line. The higher power law test p value means no significant differences from network following power law. (B) Counts and proportion of pSS DEGs in the genome-wide ceRNA network contracted. Left for lncRNA and right for coding genes. (C) Function enrichment analysis for interacting coding genes for each pSS DE lncRNA. The KEGG pathway enrichment was performed and top 20 enriched terms were shown. The dot size for counts of genes in each pathway and color density for enrichment significance as tested by hypergeometric test. pSS DE lncRNAs with significant function enrichment were shown here. [file DataSheet4.pdf]

**A**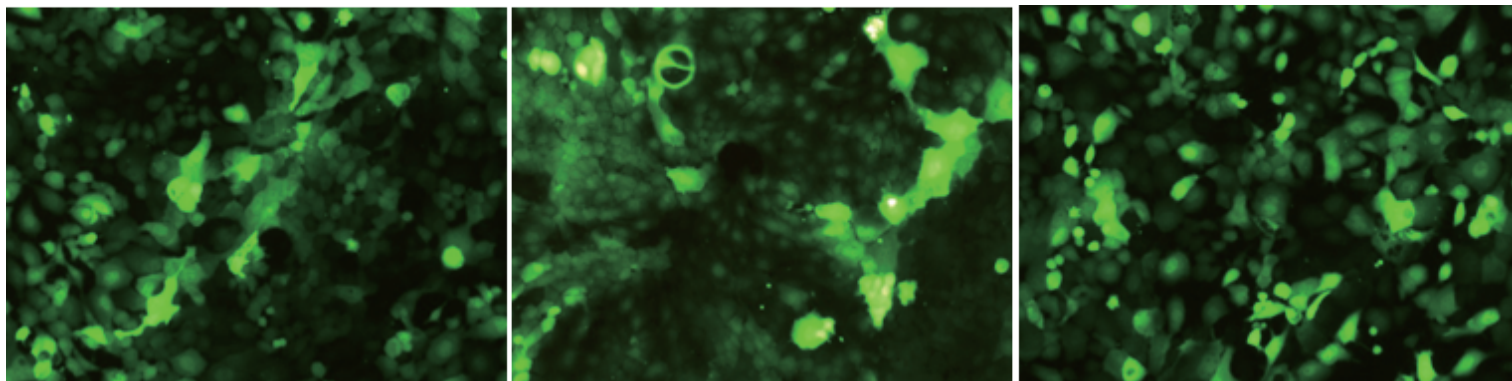**B**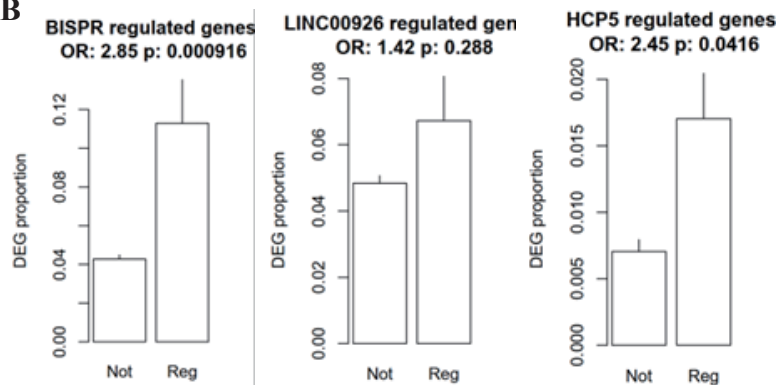**C**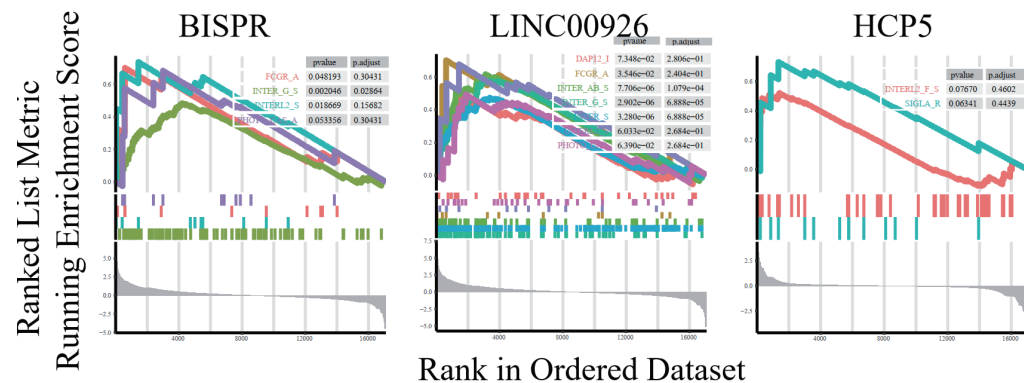**D**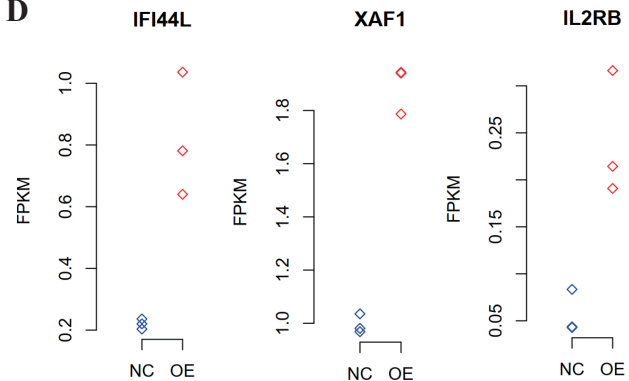**E**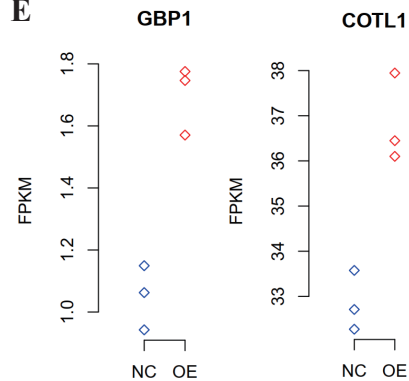**F**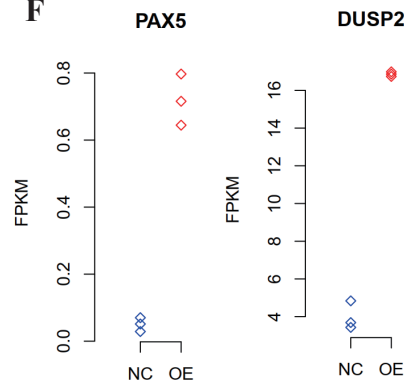

Supplement: Supplementary Figure 5 — lncRNA regulation effect validation using overexpression experiment in A253 cell lines. (A) Image of infection efficiency of the lentiviral plasmids expressing human lncRNAs into A253 cells. The 3 lncRNAs: BISPR (left), LINC00926 (middle) and HCP5 (right) were shown. (B) Proportion OE vs NC DEGs among genes interacting with lncRNA (Reg) or not (Not) in OE cell line vs NC. The OE cell lines produced for BISPR, LINC00926 and HCP5 were shown respectively. The DEG here means differentially expressed genes detected between OE and NC cell lines using RNA-seq data and the Reg means genes interacting with lncRNA based on ceRNA network constructed above in MSG samples. Not for genes not interacting with lncRNAs on ceRNA network. Y-axis for DEG proportions and error bar for 95% confidence interval. The hypergeometric test was used to compare DEG proportion differences among genes targeted or not by lncRNAs on the ceRNA network, with odds ratio and p value shown on top. (C) changes of expression of biological pathways between OE cell line and NC cell line by GSEA. X-axis for ordered ranks for genes (upper regulation in OE on left to down regulation in OE on right), and y-axis for enrichment score produced by GSEA. Top significant pathways were presented for each of 3 lncRNAs: BISPR, LINC00926 and HCP5. INTER_S, INTERFERON_SIGNALING; INTER_G_S, INTERFERON_GAMMA_SIGNALING; INTER_AB_S, INTERFERON_ALPHA_BETA_SIGNALING; DAP12_I, DAP12_INTERACTIONS; SIGLA_R, SIGNAL_REGULATORY_PROTEIN_FAMILY_INTERACTIONS; PHOTO_CAS_A, ACTIVATION_OF_THE_PHOTOTRANSDUCTION_CASCADE; PHOTO_CAS, THE_PHOTOTRANSDUCTION_CASCADE; FCGR_A, FCGR_ACTIVATION; INTERL2_S, INTERLEUKIN_2_SIGNALING; INTERL2_F_S, INTERLEUKIN_2_FAMILY_SIGNALING. (D) Expression level for 3 auto-immune disease related genes interacting with BISPR. Expression level (FPKM) in 3 lncRNA overexpression cell line (OE) samples and 3 control cell line (NC) samples were shown for each gene. The expression level difference is significant (adjusted p [file DataSheet5.pdf]
